# Cholesterol modified DP7 and pantothenic acid induce dendritic cell homing to enhance the efficacy of dendritic cell vaccines

**DOI:** 10.1186/s43556-021-00058-9

**Published:** 2021-12-05

**Authors:** Rui Zhang, Lin Tang, Qing Li, Yaomei Tian, Binyan Zhao, Bailing Zhou, Li Yang

**Affiliations:** State Key Laboratory of Biotherapy and Cancer Center, West China Hospital, Sichuan University, and Collaborative Innovation Center for Biotherapy, Chengdu, 610041 People’s Republic of China

**Keywords:** DC vaccine, DC migration, DP7-C, Pantothenic acid, miR-142a-3p

## Abstract

Dendritic cell (DC)-based cancer vaccines have so far achieved good therapeutic effects in animal experiments and early clinical trials for certain malignant tumors. However, the overall objective response rate in clinical trials rarely exceeds 15%. The poor efficiency of DC migration to lymph nodes (LNs) (< 5%) is one of the main factors limiting the effectiveness of DC vaccines. Therefore, increasing the efficiency of DC migration is expected to further enhance the efficacy of DC vaccines. Here, we used DP7-C (cholesterol modified VQWRIRVAVIRK), which can promote DC migration, as a medium. Through multiomics sequencing and biological experiments, we found that it is the metabolite pantothenic acid (PA) that improves the migration and effectiveness of DC vaccines. We clarified that both DP7-C and PA regulate DC migration by regulating the chemokine receptor CXCR2 and inhibiting miR-142a-3p to affect the NF-κB signaling pathway. This study will lay the foundation for the subsequent use of DP7-C as a universal substance to promote DC migration, further enhance the antitumor effect of DC vaccines, and solve the bottleneck problem of the low migration efficiency and unsatisfactory clinical response rate of DC vaccines.

**Supplementary Information:**

The online version contains supplementary material available at 10.1186/s43556-021-00058-9.

## Introduction

The essence of tumor immunotherapy is to prevent or treat tumors by activating the immune system or relieving the immunosuppressive state of tumors. It is considered the fourth most promising tumor treatment strategy after surgery, radiotherapy and chemotherapy. The vaccination strategy is a direct method to induce an effective immune response against cancer antigens. In most cases, vaccination against cancer antigens depends on DCs. DCs are sentinels of the immune system and initiate and guide the immune response. Since the first use of DCs loaded with melanoma-related antigens in vitro to treat melanoma in 1995, hundreds of clinical trials based on the use of DC vaccines for malignant tumors have been conducted [1]. To date, although DC-based cancer vaccines have achieved good therapeutic effects in animal experiments and early clinical trials of certain malignant tumors, the objective response rate in clinical trials rarely exceeds 15% [2].

At present, the sources and doses of antigens, antigen loading methods, DC culture methods, conditions that stimulate DC maturation limit the effectiveness of DC vaccines. Apart from the above factors, the phenotypes of the prepared DCs, number of DCs administered, routes of vaccine administration, and abilities of DCs also play crucial roles in the effective DC vaccines [3]. In recent years, how to further enhance the effect of DC vaccines in the body has received extensive attention [4, 5]. A large number of studies have focused on improving the antigen selection method, antigen loading methods, DC preparation methods, DC culture methods and vaccine administration routes to enhance the effect of DC vaccines [6–8]. However, there is still a lack of relevant research on enhancing the effect of DC vaccines by improving the ability of DCs to migrate to LNs.

Among the various functional characteristics of DCs, the endogenous migration of DCs or the ability of DCs to migrate from the injection site to draining lymph nodes (dLNs) after injection of DC vaccines prepared in vitro is critical. This process controls interactions between DCs and adaptive immune cells and activate adaptive immunity [3]. However, recent studies have found that when DC vaccines are administered, the efficiency of DC migration from the injection site to the LNs is very low, usually less than 5% [9]. The low migration rate of DC vaccines may be one of the main factors limiting the efficiency of DC vaccines. Importantly, the efficacy of DC vaccines has been shown to be closely related to the efficiency of DC migration to dLNs. Studies have shown that enhancing DC migration to dLNs can induce a stronger antitumor immune response and improve the survival rate of patients. The more DCs that migrate to dLNs, the more beneficial DC vaccines are [3, 10, 11]. Therefore, improving DC migration to dLNs can help further improve the antitumor effect of DC vaccines. Understanding and manipulating DC migration will aid in the development of new treatments and vaccination strategies. Recent research on DC migration mainly focuses on chemokines, adhesion molecules, etc., but there is a lack of general substances that can be used to promote DC migration. Therefore, it is particularly important to find a ubiquitous substance that promotes DC migration and enhances the effect of DC vaccines in the body.

In our previous studies, DP7-C, which is a cholesterol-modified derivative of antimicrobial peptides, was developed and designed by our group using computer simulations and was shown to have both delivery vehicle and immune adjuvant effects, enhancing the antitumor effect of DC vaccines loaded with lung cancer neoantigens [12, 13]. In addition, DP7-C-modified liposomes have unique advantages in mRNA delivery and can enhance the antitumor effect of mRNA vaccines encoding lung cancer neoantigens [14]. Based on the above studies, we intended to conduct clinical trials of DP7-C in combination with a DC vaccine for advanced lung cancer. However, we found that the screening period for neoantigens was too long in clinical trials of DC vaccines, which led to rapid deterioration of the disease course in some patients while waiting for neoantigens. We therefore used whole tumor lysates as antigens while waiting for neoantigens to evaluate whether DP7-C could enhance the antitumor effect of DC vaccines loaded with whole tumor lysates and determine the mechanism. The use of hypochlorous acid followed by the oxidation of tumor cells and the preparation of tumor lysates by repeated freeze-thawing has been reported to show good safety and efficacy in patients with ovarian cancer [15]. Therefore, we used hypochlorous acid (which enhances the immunogenicity of protein antigens, thereby increasing the uptake and processing of protein antigens by antigen-presenting cells, and increases the activation of antigen-specific T lymphocytes) to treat whole tumor lysates in subsequent experiments [16, 17]. The results showed that DP7-C enhances the antitumor effect of DC vaccines loaded with hypochlorite oxidation-whole tumor lysates. Thus, we sought to elucidate the mechanism by which DP7-C enhances the antitumor effect of DC vaccines loaded with whole-tumor lysates. We performed metabolomics, transcriptomics and microRNA (miRNA) sequencing after stimulation of DCs with DP7-C to identify metabolites and miRNAs that promote DC migration and to elucidate the mechanisms by which DP7-C or metabolites promote DC migration directly or through regulation of miRNAs. We further identified ubiquitous substances (migration-related metabolites) that promote DC migration and enhance the effect of DC vaccines to lay the foundation for subsequent clinical trials.

## Results

### DP7-C enhanced the antitumor effect of the DC vaccine loaded with hypochlorous acid (HOCl)-oxidized tumor cell lysates (TCLs) (HOCl-TCL) by promoting DC migration

**Fig. 1:**
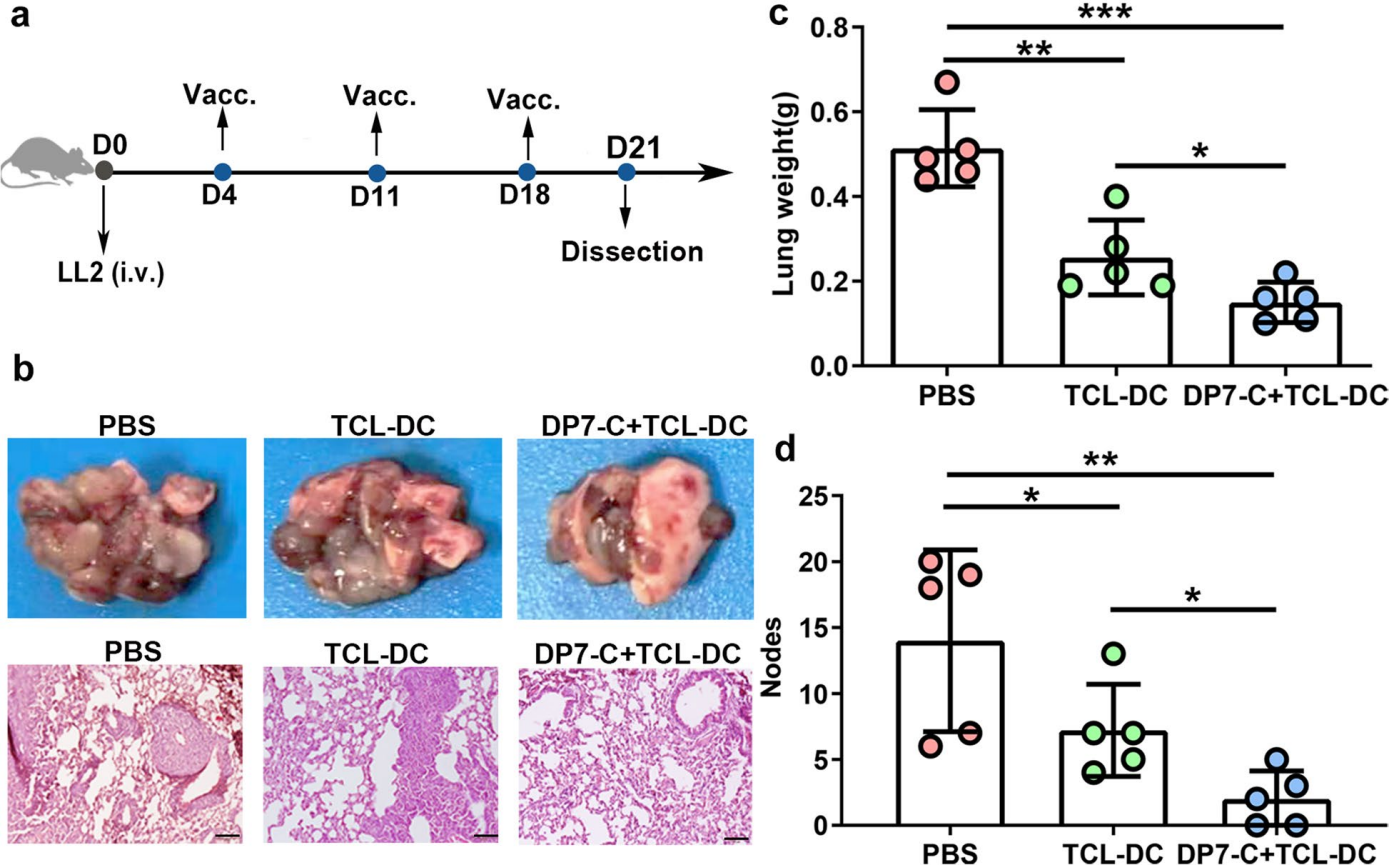
DP7-C enhances the antitumor effect of DC vaccines loaded with HOCl-oxidized TCL. C57BL/6 J mice (*n* = 5 per group) were inoculated with LL2 (3 × 10^5^). The DC vaccine was administrated three times with a 1-week interval. **a**. Diagram of the vaccine workflow. **b**. Representative results of metastatic lung nodules and HE staining results of lung tissue in each group. **c**. Lung weight in each group. **d**. Number of pulmonary nodules in each group. Scale bars, 50 μm. Significance was calculated using a one-way ANOVA with multiple comparisons tests (*p < 0.05, **p < 0.01, ***p < 0.001)

The DC vaccine loaded with HOCl-TCL has been proven to have good antitumor effects, and it can be prepared more quickly than DC vaccines loaded with neoantigens, which can save time for patients [17–19]. Our previous studies have found that DP7-C, which was independently developed and designed by our laboratory and has dual functions as a carrier and immune adjuvant, enhances the antitumor effect of the neoantigen-loaded DC vaccine [12]. In this study, we considered whether it is feasible to use DP7-C to enhance the antitumor effect of the DC vaccine loaded with HOCl-TCL. The experimental procedure was carried out as shown in Fig. 1a. The results showed that incubating DP7-C with HOCl-TCL (DP7-C/TCL-DC) could not enhance the antitumor effect of the vaccine (Supplementary Fig.1a-c), but incubating DP7-C with DCs and then adding HOCl-TCL (DP7-C + TCL-DC) could significantly improve the antitumor effect of the vaccine, which manifested as significant reductions in lung weight and the number of lung nodules (Fig. 1b-d).

**Fig. 2:**
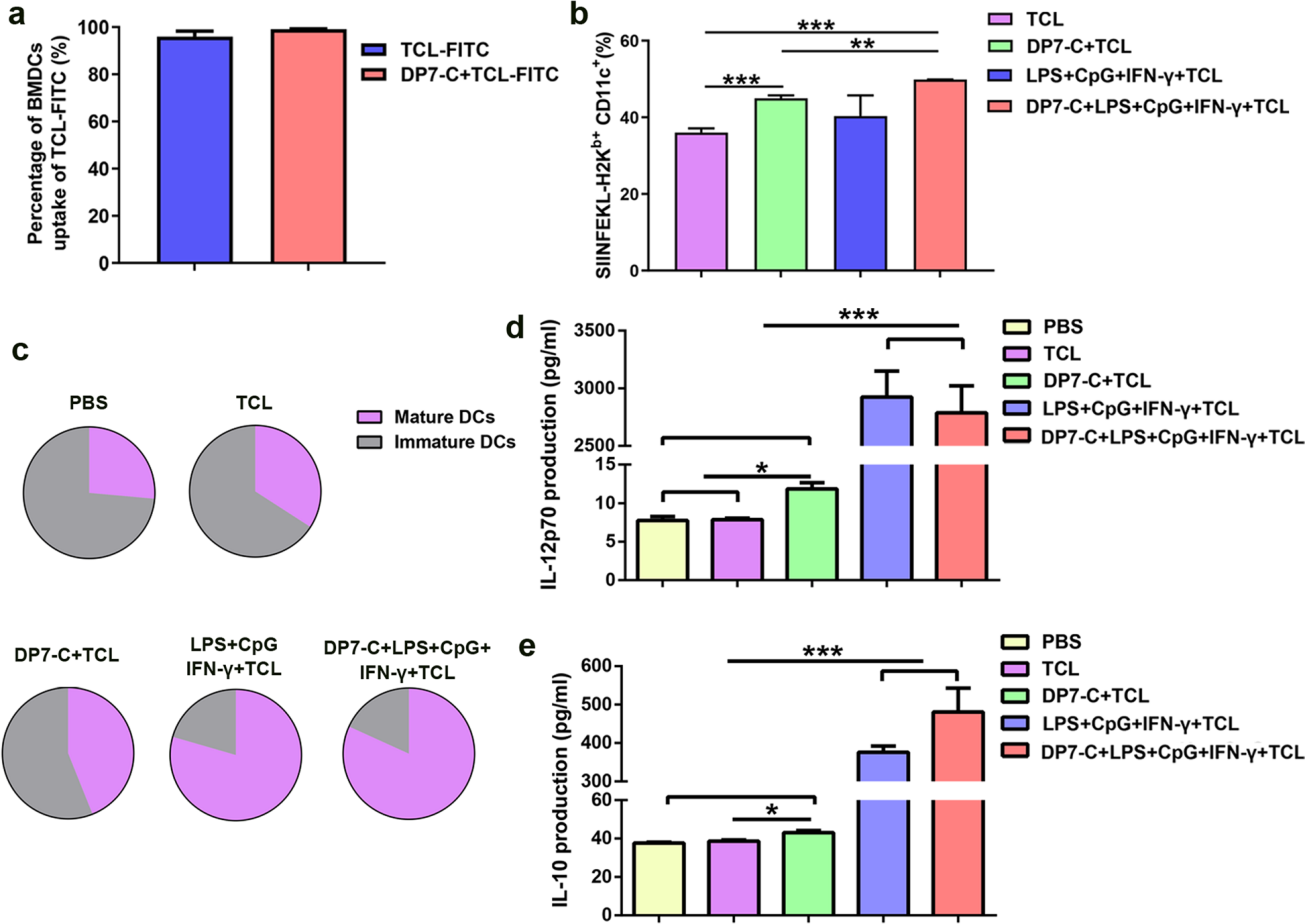
The efficiency of the antigen uptake and presentation of DCs, the maturation of DCs, and the secretion of cytokines by DCs after treatment. **a**. The uptake efficiency of DCs in FITC-labeled whole-tumor lysates. **b**. The EG7-OVA presentation efficiency of DCs from whole-tumor lysates stained with the monoclonal antibody 25-D1.16, which recognizes the OVA_257–264_-H-2Kb complex. **c**. Percentage of mature DCs among all DCs after different treatments. **d**. Detection of IL-12p70 in culture supernatants by ELISA after different treatments. **e**. Detection of IL-10 in culture supernatants by ELISA after different treatments. Significance was calculated using a one-way ANOVA with multiple comparisons tests. (*p < 0.05, **p < 0.01, ***p < 0.001)

**Fig. 3:**
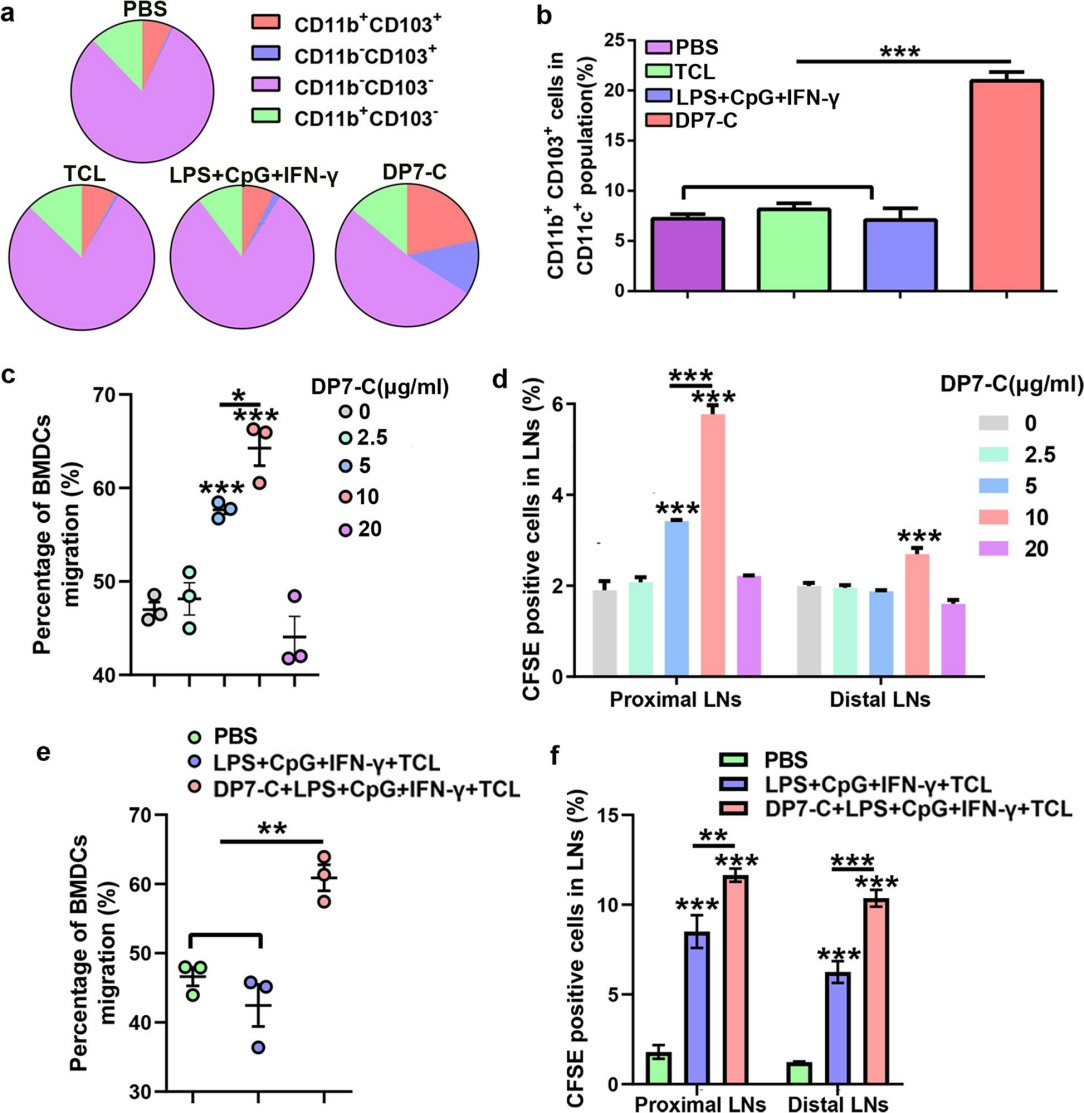
DP7-C enhances DC migration in vivo and in vitro. **a-b**. Flow cytometry was performed to detect the percentage of CD103^+^ DCs among all DCs after different treatments. **c**. Transwell assays showed that DP7-C enhanced the migration of DC in vitro (the lower chambers contained 500 μl of RPMI 1640 medium with 10% FBS + CCL19 (250 ng/ml) + CCL21 (250 ng/ml))*.* **d**. Flow cytometry revealed that DP7-C enhanced the LNs homing of CFDA-SE labeled DCs (*n* = 3). **e**. Transwell assays were used to detect the migration of DCs in vitro after different treatments. **f**. Flow cytometry detection of different treatments and their effects on the LN homing of CFDA-SE labeled DCs (*n* = 3). Proximal LNs were the inguinal lymph node on the same side as the DCs injection, and Distal LNs were the inguinal lymph node on the other side from the DCs injection. Significance was calculated using a one-way ANOVA with multiple comparisons tests. (*p < 0.05, **p < 0.01, ***p < 0.001)

To further explore the mechanism by which DP7-C enhances the antitumor effect of DC vaccines loaded with HOCl-TCL, we first tested whether DP7-C plays the role of a delivery vehicle. The results showed that when the OVA_257–264_ concentration was low, DP7-C pretreatment and the combination of lipopolysaccharide (LPS) + CpG + IFN-γ (LCI) could significantly improve the efficiency of antigen presentation (Supplementary Fig. 2b-c). When the OVA_257–264_ concentration was high or LL2-TCL-FITC was applied, there was no significant difference in the antigen uptake efficiency of DCs after DP7-C pretreatment (Fig. 2a, Supplementary Fig. 2d-f). The presentation efficiency was significantly increased after DP7-C treatment, but there was no significant difference in the antigen presentation efficiency after application of the combination of LCI (Fig. 2b, Supplementary Fig. 2d-f). Moreover, we found that the addition of DP7-C to the final vaccine formulation at high antigen concentrations did not affect the average fluorescence intensity reflecting antigen uptake and presentation (Supplementary Fig. 2a, c, d, f). Next, we assessed whether DP7-C could exert an immune adjuvant effect. The results showed that there was no significant difference between the DP7-C + LCI + TCL group and the LCI + TCL group in terms of promoting DC maturation and cytokine secretion (Fig. 2c-e, Supplementary Fig. 3a-d). In previous studies, we also found that DP7-C treatment increased the proportion of CD103^+^ DCs (a typology that promotes DC migration) and the efficiency of DC migration to LNs [12, 14]. Therefore, we further verified the efficacy of DP7-C in promoting DC migration. The results showed that DP7-C promoted DC migration in vivo and in vitro in a concentration-dependent manner (Fig. 3a-f, Supplementary Fig. 3e).

### DP7-C and the metabolite PA promoted DC migration by upregulating the chemokine receptor CXCR2

**Fig. 4:**
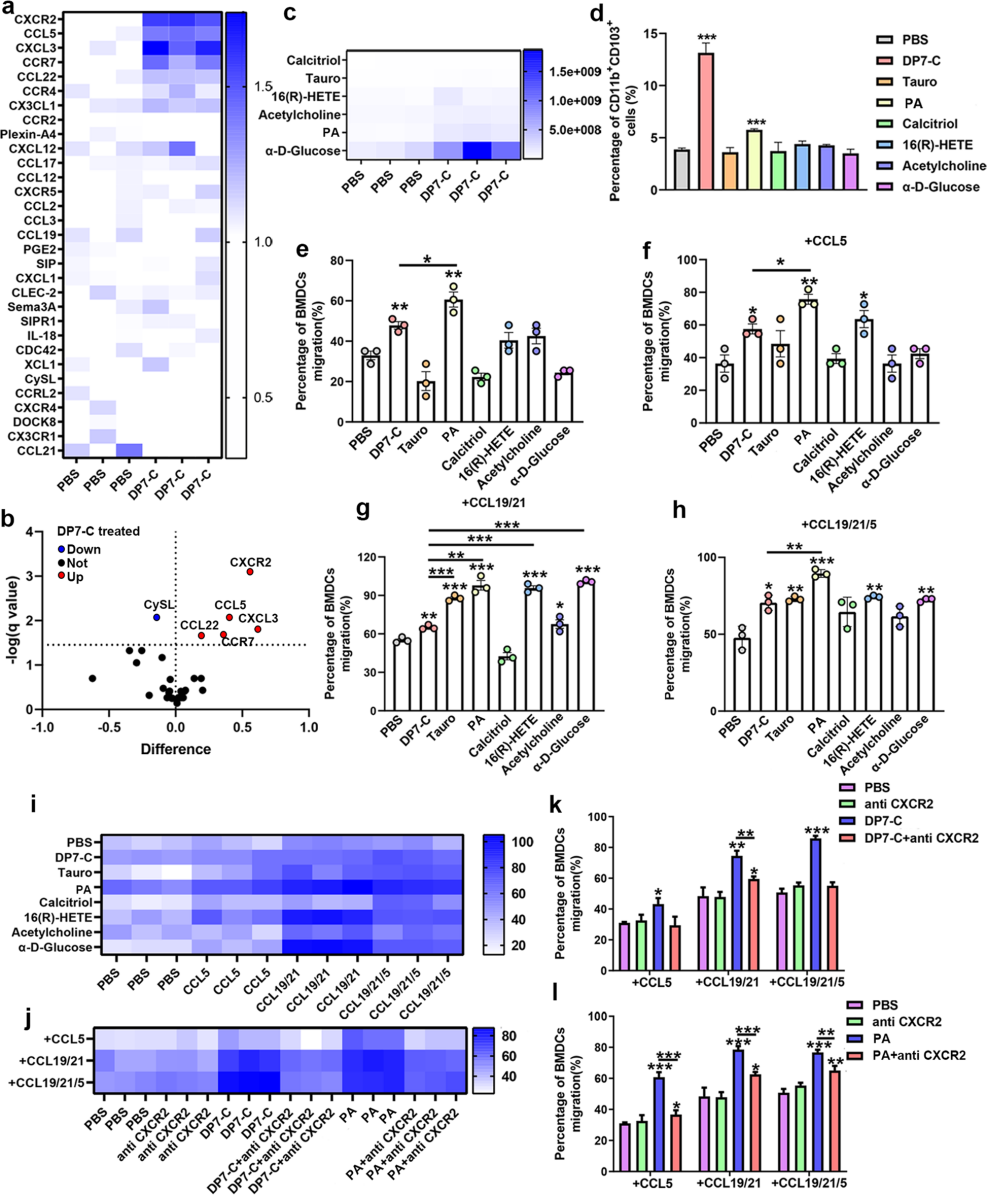
DP7-C and PA promote DC migration by activating CXCR2. **a-b**. RT-PCR was used to detect the expression of chemokines and their receptors in DCs treated with DP7-C. **c**. Metabolomics sequencing detected that DP7-C increased the content of migration-related metabolites in DCs. **d**. Flow cytometry was performed to detect the percentage of CD103^+^ DCs among all DCs after metabolite treatment. **e-i**. Transwell assays were used to detect the efficiency of DC migration in vitro after different treatments. **j-l**. Pretreatment of DC with CXCR2 antibody inhibited DP7-C and PA-mediated DC migration in vitro. Significance was calculated using a one-way ANOVA with multiple comparisons tests (*p < 0.05, **p < 0.01, ***p < 0.001)

Chemokines and their receptors are the keys to cell migration, so we first detected the expression of various common chemokines and their receptors after DP7-C treatment. The results showed that the expression levels of CCR7, CCL5, CCL22, CXCL3 and CXCR2 were obviously upregulated in treated versus untreated cells, and the change in CXCR2 was the most obvious (Fig. 4a-b). Therefore, we speculated that the promotion of DC migration in vitro by DP7-C might be controlled by CXCR2. To further determine the key metabolites controlling DC migration, we performed metabolomics sequencing and identified the major metabolites of DCs that changed obviously after DP7-C treatment. There were 101 upregulated metabolites and 183 downregulated metabolites in treated versus untreated cells (Supplementary Fig. 4a-d). Through screening and analysis of all the differentially expressed metabolites, we identified six metabolites that have been reported to have the ability to regulate the migration of other cells, namely, calcitriol, taurochenodeoxycholic acid (Tauro), 16(R)-HETE, acetylcholine, PA and α-D-glucose-1,6-bisphosphate (α-D-Glucose), for use in subsequent experiments (Fig. 4c). First, we assessed the ratio of CD103^+^ DCs after metabolite treatment and found that the ratio of CD103^+^ DCs only increased after PA treatment (Fig. 4d). Next, we assessed the expression of several chemokines and receptors that were upregulated after DP7-C treatment in DCs after metabolite treatment. We found that the expression of CXCR2 in DCs was upregulated after Tauro, 16(R)-HETE and PA treatment. CXCL3 expression was upregulated in DCs after Tauro and PA treatment (Supplementary Fig. 5a-f). We further assessed the promoting effects of DP7-C and its metabolites on DC migration in vitro. By adding or not adding various cytokines to the lower chamber medium for the migration assay, we found that only PA was better than DP7-C in promoting DC migration in each group (Fig. 4e-i). Therefore, we believe that PA is a key metabolite by which DP7-C regulates DC migration and that PA might be used as a ubiquitous metabolite that induces DC migration to LNs to enhance the antitumor effect of DC vaccines. Then, we verified the mechanism by which DP7-C and PA promote DC migration and found that the promotion of DC migration by DP7-C and PA was inhibited to a certain extent by an anti-CXCR2 antibody (Fig. 4j-l), indicating that CXCR2 is indeed the key receptor of DP7-C and that PA promotes DC migration.

### Transcriptomics and miRNA sequencing data indicated that DP7-C may regulate miRNAs and affect the NF-κB signaling pathway, thus regulating DC migration

**Fig. 5:**
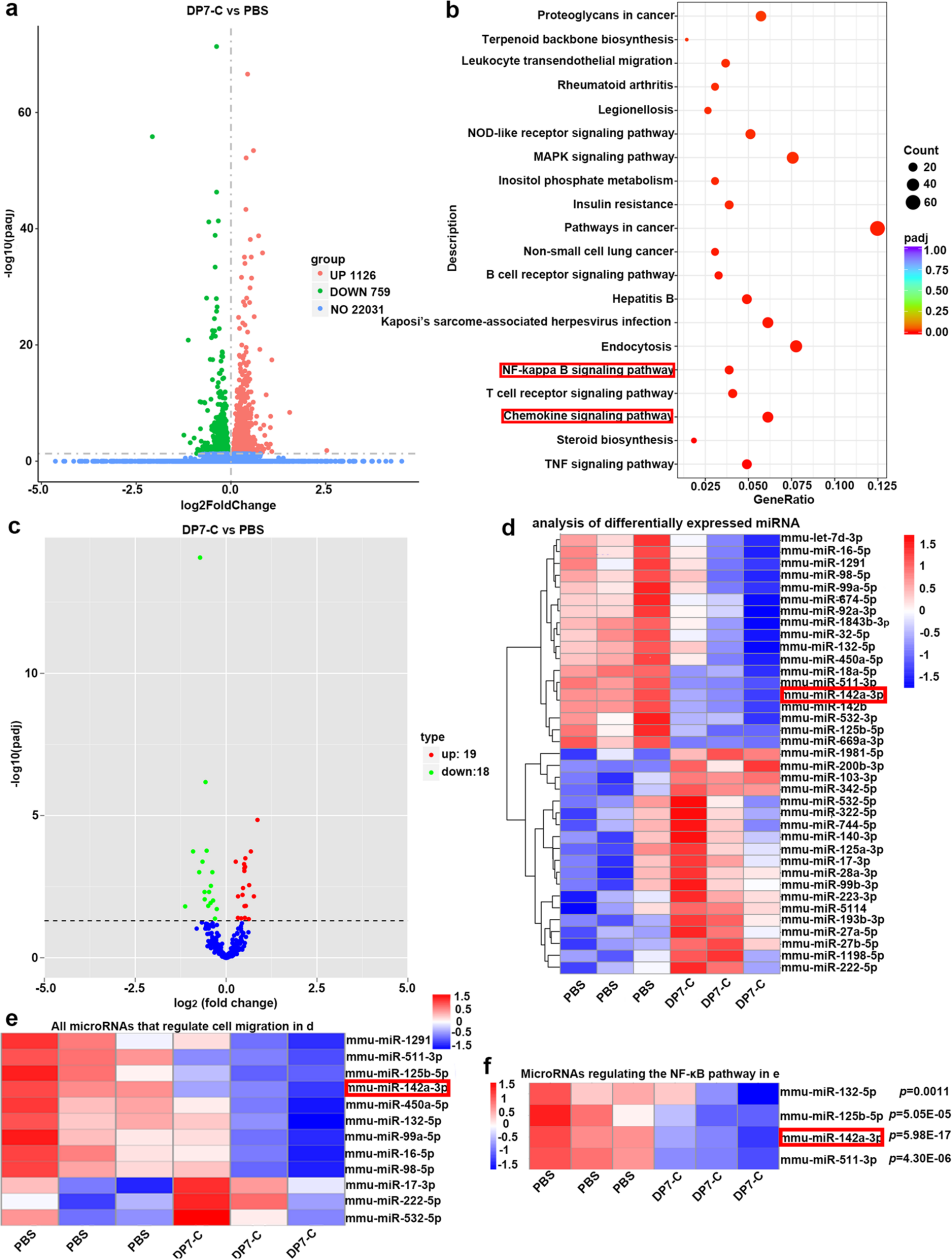
Transcriptomics and miRNA sequencing results after DP7-C treatment of DCs. **a**. Volcano map of differentially expressed genes in DCs before and after DP7-C treatment. **b**. KEGG pathway enrichment analysis of differentially expressed genes. **c**. KEGG pathway enrichment analysis of differentially expressed genes. **d**. Cluster analysis of differentially expressed miRNAs. **e**. Heat map of miRNAs that regulate cell migration. **f**. Heat map of miRNAs that regulate the NF-κB signaling pathway

DC migration induced by DP7-C and PA was not completely inhibited after applying the anti-CXCR2 antibody, indicating that there are other mechanisms by which DP7-C and PA promote DC migration. Therefore, we performed transcriptomics sequencing and miRNA sequencing of DCs after DP7-C treatment. We analyzed the pathway enrichment of genes altered at the transcriptome level, and we found that the migration-related chemokine signaling pathway and NF-κB signaling pathway were obviously upregulated after DP7-C treatment (Fig. 5a-c). We analyzed the sequencing data of miRNAs, and we found that 19 miRNAs were upregulated and 18 miRNAs were downregulated after DP7-C treatment (Fig. 5d). After screening all the miRNAs, we found that 9 downregulated miRNAs and 3 upregulated miRNAs were reported to have the ability to promote the migration of other cells (Fig. 5e) [20–31]. Further analysis revealed that 4 downregulated miRNAs were reported to regulate the NF-κB signaling pathway (Fig. 5f) [22, 32–34]. In addition, we found that miR-142a-3p was effective in regulating cell migration and the NF-κB signaling pathway, and it had the smallest *p* values among all the changed miRNAs. Therefore, we speculated that DP7-C and PA might regulate the NF-κB signaling pathway by regulating miR-142a-3p, thus affecting DC migration.

### DP7-C and PA promote DC migration by inhibiting miR-142a-3p, thus activating the NF-κB signaling pathway in vitro

**Fig. 6:**
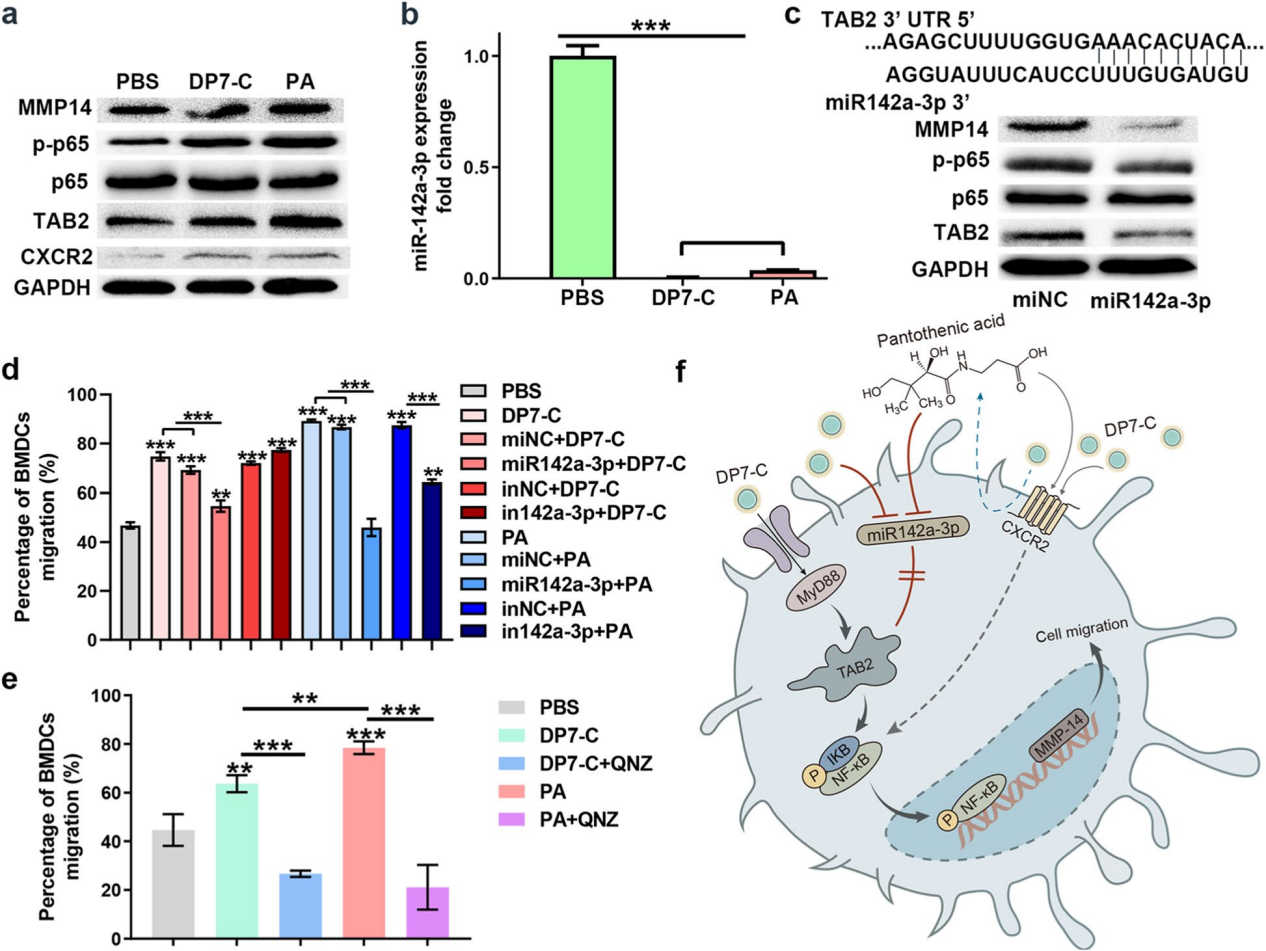
DP7-C and PA can promote DC migration by inhibiting miR-142a-3p, thus activating TAB2 and the NF-κB signaling pathway. **a**. Western blot experiments revealed that DP7-C and PA activated the expression of CXCR2, TAB2, NF-κB p-p65 and MMP14 in DCs. **b**. RT-PCR revealed that the expression of miR-142a-3p in DCs was significantly inhibited after DP7-C and PA treatment. **c**. Western blot experiments revealed that pretransfection of cell with miR-142a-3p inhibited the expression of TAB2, NF-κB p-p65 and MMP14. **d**. Transwell experiments revealed that pretransfection of cells with miR-142a-3p inhibited DP7-C and PA-mediated cell migration in vitro. **e**. Western blot experiments revealed that pretreatment of cells with NF-κB inhibitor (QNZ) in vitro inhibited DP7-C- and PA-mediated cell migration. **f**. Proposal of mechanism by which DP7-C promotes DC migration. Significance was calculated using a one-way ANOVA with multiple comparisons tests (*p < 0.05, **p < 0.01, ***p < 0.001)

Next, we aimed to verify whether DP7-C and PA can promote NF-κB signaling and DC migration by inhibiting miR-142a-3p. First, through western blotting experiments, we detected that DP7-C and PA can upregulate the target gene of miR-142a-3p, TAB2, and the expression of NF-κB p65 and p-p65 (Fig. 6a). Second, through RT-PCR experiments, we detected that the expression of miR-142a-3p was significantly downregulated in DCs treated with DP7-C and PA (Fig. 6b), while transfection of miR-142a-3p mimics significantly inhibited TAB2 and NF-κB p-p65 expression and inhibited DC migration in vitro (Fig. 6c-d). After treating DCs with the NF-κB inhibitor QNZ, the induction of cell migration by DP7-C and PA was completely inhibited (Fig. 6e), indicating that DP7-C and PA can indeed promote NF-κB signaling by inhibiting the expression of miR-142a-3p, which in turn promotes DC migration (Fig. 6f).

### Study on the mechanism of DP7-C and PA in promoting DC migration to dLNs in vivo

**Fig. 7:**
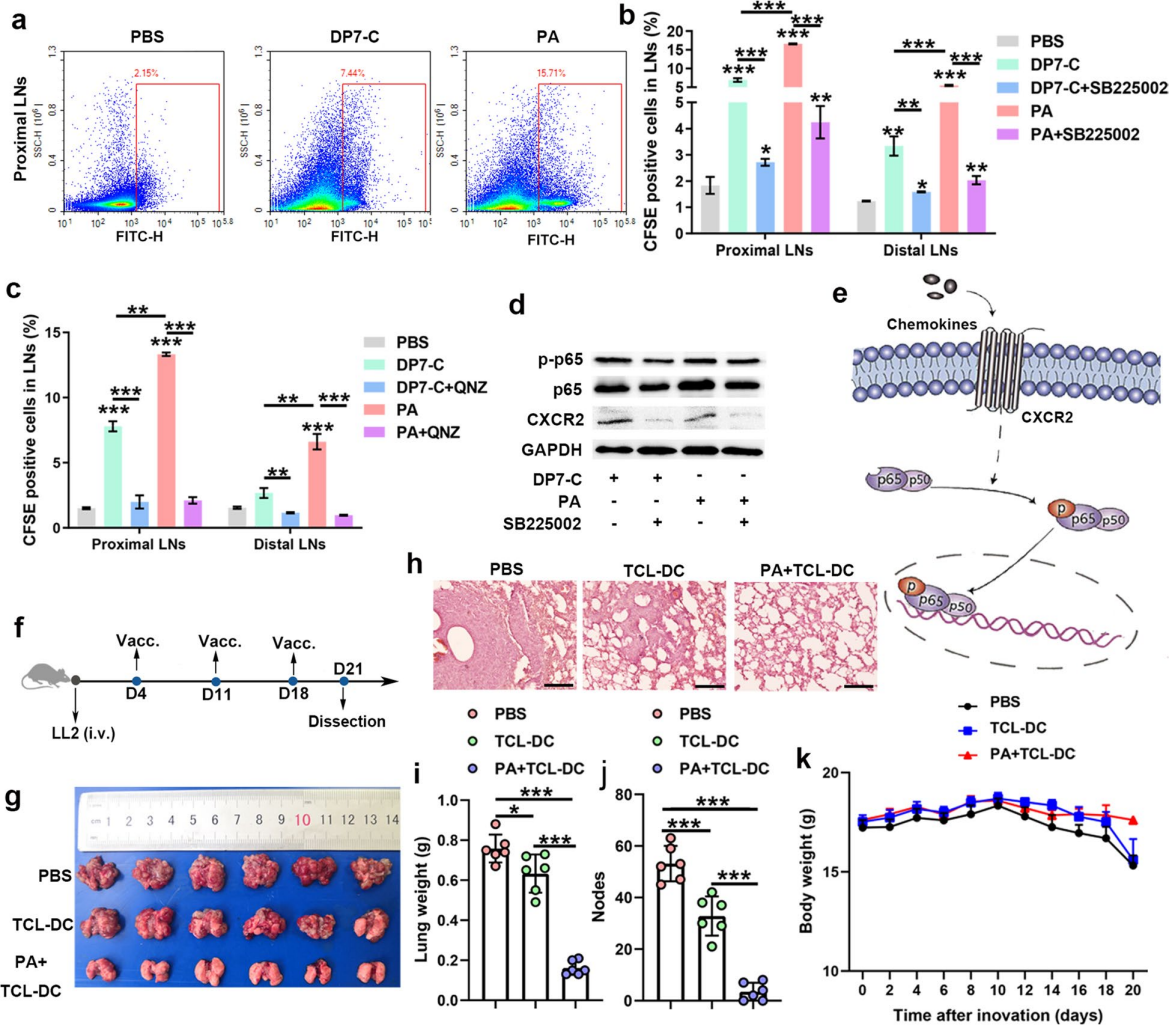
DP7-C and PA enhance the effect of DC vaccines by promoting DC migration to LNs. **a**. The histogram of the flow cytometry results showing that PA enhances the LNs homing of CFDA-SE labeled DC in vivo. **b**. Preadministration of an inhibitor of CXCR2 in vivo decreased the efficiency of DP7-C- and PA-mediated LN homing in DCs (*n* = 3). **c**. Preadministration of an inhibitor of NF-κB in vivo inhibited the efficiency of DP7-C- and PA-mediated LN homing in DCs (*n* = 3). **d-e**. Western blotting revealed that pretreatment of cells with a CXCR2 inhibitor can inhibit the expression of NF-κB p65 and p-p65. **f**. Diagram of the vaccine workflow. **g**. Representative results of metastatic lung nodules. **h**. HE staining results of lung tissue in each group. Scale bars, 50 μm. **i**. Lung weight in each group. **j**. Number of pulmonary nodules in each group. **k**. Mouse body weight changes during treatment in each group. Significance was calculated using a one-way ANOVA with multiple comparisons tests (*p < 0.05, **p < 0.01, ***p < 0.001)

To further verify whether we could inhibit DP7-C- and PA-induced DC migration to LNs, we intraperitoneally injected SB225002 (an inhibitor of CXCR2 [35]) and QNZ (an inhibitor of NF-κB) into mice and then tested the effect on DC migration to LNs. The results showed that DC migration to LNs was inhibited after treatment with SB225002 but was still higher than that in the control group (Fig. 7a-b), while DC migration to LNs was completely inhibited after treatment with QNZ (Fig. 7c). In addition, we verified that NF-κB was inhibited after CXCR2 inhibition, indicating that CXCR2 also acts by activating the downstream NF-κB signaling pathway (Fig. 7d-e).

### PA enhanced the antitumor effect of DC vaccines loaded with HOCl-TCL

To verify whether PA can enhance the antitumor effect of the DC vaccines loaded with HOCl-TCL, an experiment was carried out according to the process in Fig. 7f. The results showed that the antitumor effect increased significantly after PA was added, and this effect was reflected by the significant decreases in the lung weight and the number of pulmonary nodules in the PA group versus the other control groups (Fig. 7g-j). Finally, we evaluated the safety of the combination of the DC vaccine with PA by recording the weights of the mice during vaccine treatment and by HE staining of the main organs of the mice after vaccine treatment. The results showed that the weight of mice during treatment did not decrease significantly (Fig. 7k), and no obvious toxicity or side effects were found in the organs of the mice after vaccine treatment (Supplementary Fig. 6), indicating that the combination of the DC vaccine with PA was safe. In addition, none of the final vaccine formulations caused significant cytotoxicity to DC (Supplementary Fig. 7a). Furthermore, after DP7-C and PA were added to the DC vaccine formula, the main types of DC were CD4^+^CD8^−^DC (cDC2), langerhans cell (LC), monocyte derived DC (Mo-DC) and CD8^+^ DC (cDC1) (Supplementary Fig. 7b-c).

## Discussion

At present, there are three main ways to increase DC migration to dLNs: applying proinflammatory mediators or stress-inducing pretreatment, increasing the ability of dLNs to recruit DCs, and influencing DC migration by influencing the maturation of DCs. First, pretreatment with proinflammatory mediators or stress-inducing factors can enhance DC migration to dLNs. Proinflammatory mediators mainly play a role by inducing DCs to mature. These mediators usually affect DC migration in two ways: 1) by directly affecting DC maturation and inducing migration to dLNs or 2) by the production of local cytokines, including (but not limited to) chemokines that induce DC migration and recruit circulating immune cells, such as IL-1β, TNF-α and prostaglandin E2, which have been proven to be key to initiating DC migration [36]. However, the influence of cytokines on DC migration largely depends on the dose. It has been reported that a low concentration of TNF-α (e.g., ~ 50 U/ml) can promote cell migration, while a high concentration of TNF-α (e.g., ~ 5000 U/ml) can inhibit cell migration [37]. This phenomenon was also observed in our experiments, that is, a low concentration of DP7-C promoted DC migration, while a high concentration of DP7-C inhibited DC migration (Fig. 3c-d). The dependency of DC migration on the dose of recruited factors probably holds true for other factors as well, but this obviously requires further investigation. Other studies have found that skin stimulation and laser irradiation can improve the efficiency of DC migration to dLNs, which indicates that stress reactions may contribute to DC migration [38]. This phenomenon and its mechanism need further study, so it cannot currently be used as a general method to stimulate DC migration. Increasing the ability of dLNs to recruit DCs is another way to increase DC migration. This strategy mainly involves enhancing the ability of LNs to recruit DCs by using NK or CD4^+^ T cells residing in LNs and increasing the efficiency of DC migration by using lymphotoxin, IL-7 and CCL21 to induce changes in ectopic lymphatic tissue [39–41]. However, these methods often involve the recruitment of specific cell types, such as CD83^+^CCR7^+^ NK cells, and the process is complicated, so they cannot be used as a general method to enhance DC migration at present. In addition, inducing the maturation of injected immature DCs allows them to migrate to dLNs. Studies have shown that local application of imiquimod cream can significantly improve DC migration, while intradermal injection of soluble imiquimod or TLR7/8 ligand R848 does not have the same effect [42]. In another study, TLR4 natural ligand promoted the maturation of DCs, enhanced the expression of CCR7 on DC and enhanced the migration ability of DCs to dLNs [43]. Therefore, the mature state and process of DCs are very important for DC migration, and their specific mechanisms and application methods need further study.

Migration from nonlymphoid tissue to lymphoid tissue is the key feature of DCs. The migration ability of DCs is key to triggering a protective proinflammatory response and tolerant immune response. Understanding and manipulating DC migration will aid in the development of new treatment and vaccination strategies. At present, research on DC migration mainly focuses on chemokines, adhesion molecules, etc., but there is a lack of knowledge regarding ubiquitous substances that can be used to promote DC migration [44, 45]. Therefore, it is particularly important to find ubiquitous substances to promote DC migration and enhance the effect of DC vaccines in vivo. Studies have shown that metabolites can promote DC migration and enhance the antitumor effect of DC vaccines [46], which suggests that we may be able to enhance the effect of DC vaccines by looking for metabolites related to DC migration. Therefore, in this study, DP7-C, which promotes DC migration, was used as the medium. Multiomics sequencing and biological experiments were performed to determine the general metabolites that enhance DC migration and verify the specific mechanism by which they promote DC migration, providing experimental and theoretical bases for further enhancing the effect of DC vaccines by increasing the efficiency of DC migration to dLNs.

This study clarified the mechanism by which DP7-C enhances the effect of DC vaccines by enhancing DC migration and revealed that the metabolite PA has the same effect. However, other metabolites that we found in this experiment, such as calcitriol, Tauro, 16(R)-HETE, acetylcholine, and α-D-glucose, also promoted DC migration in vitro. We only selected PA for follow-up experiments and did not consider whether the combination of multiple metabolites in treating DCs could achieve better promotion of DC migration and enhance the antitumor effect of the DC vaccine. Therefore, in follow-up experiments, we will use a variety of metabolites to further improve the antitumor effect of DC vaccines by increasing the efficiency of DC migration. In addition, we found that both DP7-C and PA induced CD103^+^ DC production, and CD103^+^ DCs were reported to be closely associated with DC migration [47]. In the present study, we did not delve further into the possible relationship between CXCR2 expression and CD103^+^ DCs, and we will continue to investigate this manifestation further in subsequent studies.

In conclusion, based on previous studies, DP7-C, which can promote DC migration, was used as a medium. We screened out the metabolite PA by multiomics sequencing and biological experiments and determined that it can also promote DC migration to LNs. This study verified that DP7-C and PA can enhance the antitumor effect of DC vaccines by upregulating CXCR2 and inhibiting miR-142a-3p, thereby activating the NF-κB signaling pathway to promote DC migration. Both DP7-C and PA can be used as ubiquitous substances to promote DC migration, which lays a foundation for subsequent clinical trials.

## Materials and methods

### Cells and animals

Roswell Park Memorial Institute (RPMI) 1640 medium and Dulbecco’s modified Eagle’s medium (DMEM) containing 100 units/ml streptomycin and penicillin (PS) and 10% fetal bovine serum (FBS) were used to culture bone marrow-derived DCs (BMDCs), LL2 cells and EG7-OVA cells (American Type Culture Collection, Manassas, VA, USA). All cells were cultured in a cell incubator containing 5% CO_2_ at 37 °C. RPMI 1640, DMEM, FBS and PS were all purchased from Thermo Fisher Scientific. Female six- to eight-week-old C57BL/6 J mice were purchased from HFK Bioscience (Beijing, China).

BMDCs were obtained from 4- to 6-week-old C57BL/6 J female mice according to a previously reported protocol [48]. Briefly, after treating bone marrow cells with red blood cell lysis buffer, fresh RPMI 1640 complete medium containing 20 ng/ml granulocyte-macrophage colony-stimulating factor (GM-CSF, PrimeGene Biotechnology, Shanghai, China) was added to 3 × 10^6^ mouse bone marrow cells. At day 8, the BMDCs were collected for further use.

### Preparation of HOCl-TCL

We followed the protocol from a previous report to prepare HOCl-oxidized LL2 and EG7-OVA lysate [18]. Briefly, NaOCl reagent (Sigma-Aldrich) was diluted with DPBS (Dulbecco’s Phosphate Buffered Saline, Cellgro) and immediately added to the tumor cells to prepare a 0.7 μM HOCl solution. The final cell density was 1 × 10^6^ cells/ml in DPBS. The tumor cell suspensions were incubated at 37 °C for 1 h with gentle agitation every 30 min to induce oxidation-dependent tumor cell death. At the end of the 1 h treatment, the tumor cells were harvested, washed twice with DPBS (3 times the volume of DPBS was added to each volume of HOCl solution to ensure complete removal of HOCl), and subjected to 7 freeze-thaw cycles. For this, the TCLs were frozen in liquid nitrogen for ≥5 min and thawed completely at room temperature 7 times to completely rupture the tumor cells.

### Flow cytometry assay

To detect the efficiency of antigen uptake and presentation, DCs were incubated with DP7-C (10 μg/ml), lipopolysaccharide (LPS; 1 μg/ml; Beyotime Biotechnology, Shanghai, China) + CpG (CpG 1826 oligonucleotide, 10 μg/ml; Invitrogen, Carlsbad, CA, USA) + IFN-γ (50 ng/ml; PrimeGene Biotechnology, Shanghai, China), or DP7-C (10 μg/ml) + LPS (1 μg/ml) + CpG (10 μg/ml) + IFN-γ (50 ng/ml) for 1 h, and then fluorescein isothiocyanate (FITC)-labeled OVA_257–264_ (2 μg/ml and 20 μg/ml) or LL2-TCL or EG7-OVA-TCL were added to the sample for another 24 h. Finally, the antigen uptake and presentation efficiency of the DCs were detected by staining with anti-mouse CD11c-APC antibody and the 25-D1.16-PE monoclonal antibody (BD, US) for 40 min followed by flow cytometry. The control group was treated with FITC-OVA_257–264_ (2 μg/ml and 20 μg/ml) or FITC-TCL. All tests were repeated three times.

To detect DC maturation after treatment, DCs (3 × 10^5^/ml) were treated with TCL (3 × 10^5^ cells), DP7-C (10 μg/ml) + TCL (3 × 10^5^ cells), TCL (3 × 10^5^ cells) + LPS (1 μg/ml) + CpG (10 μg/ml) + IFN-γ (50 ng/ml), or DP7-C (10 μg/ml) + TCL (3 × 10^5^ cells) + LPS (1 μg/ml) + CpG (10 μg/ml) + IFN-γ (50 ng/ml) for 24 h, followed by staining with anti-mouse-CD11c-APC, anti-mouse-CD86-Percp, and anti-mouse-CD80-FITC fluorescent antibodies (BD, US) for 40 min. Then, the proportion of mature DCs (CD11c^+^CD80^+^CD86^+^ DCs) was detected by flow cytometry. All tests were repeated three times.

To detect the proportion of CD103^+^ DCs, DCs (3 × 10^5^/ml) were treated with PBS, TCL (3 × 10^5^ cells), DP7-C (10 μg/ml), LPS (1 μg/ml) + CpG (10 μg/ml) + IFN-γ (50 ng/ml), Calcitriol (10 nM), taurochenodeoxycholic acid (Tauro, 50 μM), 16(R)-HETE (0.5 μM), acetylcholine (1 μM), pantothenic acid (PA, 1 mM), or α-D-glucose-1,6-bisphosphate (α-D-Glucose, 15 μM) for 24 h and the stained with anti-mouse CD11c-APC, anti-mouse CD11b-FITC and anti-mouse CD103-PE fluorescent antibodies (BD, US) for 40 min. Then, the proportion of CD103^+^ DCs was detected by flow cytometry. All tests were repeated three times. All of the gating strategy and representative diagram are placed in Supplementary Fig. 2 g.

### Cytokine detection

The supernatants of DCs (3 × 10^5^/ml) treated with TCL (3 × 10^5^ cells), DP7-C (10 μg/ml) + TCL (3 × 10^5^ cells), TCL (3 × 10^5^ cells) + LPS (1 μg/ml) + CpG (10 μg/ml) + IFN-γ (50 ng/ml), or DP7-C (10 μg/ml) + TCL (3 × 10^5^ cells) + LPS (1 μg/ml) + CpG (10 μg/ml) + IFN-γ (50 ng/ml) were diluted in a gradient, and the levels of IL-12p70 and IL-10 were detected by ELISA kits (Novus) according to the vendor’s instructions. All tests were repeated three times.

### Cell migration assay

In the in vitro migration experiments, DCs (3 × 10^5^/ml) treated with DP7-C (0, 2.5, 5, 10, 20 μg/ml), TCL (3 × 10^5^ cells) + LPS (1 μg/ml) + CpG (10 μg/ml) + IFN-γ (50 ng/ml), or DP7-C (10 μg/ml) + TCL (3 × 10^5^ cells) + LPS (1 μg/ml) + CpG (10 μg/ml) + IFN-γ (50 ng/ml) for 24 h were collected and transferred to a corning costar transwell plate (pore size: 5.0 μm; diameter: 6.5 mm). The upper chamber contained 1 × 10^5^/cells in 100 μl RPMI 1640 media. The lower chamber contained 500 μl of RPMI 1640 medium with 10% FBS + CCL19 (250 ng/ml) + CCL21 (250 ng/ml). Cells remaining in the upper chamber were wiped off after 24 h, and the migrated cells in the lower chamber were counted.

In the in vitro migration experiments, DCs (3 × 10^5^/ml) treated with DP7-C (10 μg/ml), calcitriol (10 nM), Tauro (50 μM), 16(R)-HETE (0.5 μM), acetylcholine (1 μM), PA (1 mM), and α-D-glucose (15 μM) for 24 h were collected and transferred to a transwell plate. The upper chamber contained 1 × 10^5^/cells in 100 μl RPMI 1640 media. The lower chambers contained 500 μl of RPMI 1640 medium with 1) 10% FBS, 2) 10% FBS + CCL5 (250 ng/ml), 3) 10% FBS + CCL19 (250 ng/ml) + CCL21 (250 ng/ml), or 4) 10% FBS + CCL5 (250 ng/ml) + CCL19 (250 ng/ml) + CCL21 (250 ng/ml). Cells remaining in the upper chamber were wiped off after 24 h, and the migrated cells in the lower chamber were counted. CXCR2 activity was inhibited by treating cells with mouse anti-CXCR2 (1 μg/ml, Absin) for 1 h before the migration assay [46]. NF-κB activity was inhibited by treating the cells with QNZ (10 nM, Selleckchem, US) for 1 h before the migration assay [12]. To verify whether the high expression of miR-142a-3p can inhibit DP7-C- and PA-induced DCs migration, we used Lipo3000 (0.48 μg, Invitrogen) to transfect DCs (5 × 10^5^) with miR-142a-3p mimics (miR142a-3p, 0.24 μg) or miR-142a-3p inhibitor (in142a-3p, 0.24 μg) for 4 h and then added DP7-C (10 μg/ml) and PA (1 mM) for another 20 h before performing the migration experiments. All tests were repeated three times.

In the in vivo migration experiments, DCs (3 × 10^5^/ml) treated with PBS, DP7-C (0, 2.5, 5, 10, 20 μg/ml), TCL (3 × 10^5^ cells) + LPS (1 μg/ml) + CpG (10 μg/ml) + IFN-γ (50 ng/ml), DP7-C (10 μg/ml) + TCL (3 × 10^5^ cells) + LPS (1 μg/ml) + CpG (10 μg/ml) + IFN-γ (50 ng/ml), or PA (1 mM) for 24 h were collected and labeled with carboxyfluorescein diacetate succinimidyl ester (CFDA-SE) (Beyotime Biotechnology, Shanghai, China) according to the instructions of the kit. The labeled DCs were then injected into the hind foot pads of mice (1 × 10^6^/50 μl). After 24 h, the dLNs (inguinal lymph nodes) were separated, and the cells in the dLNs were collected by picking with a needle. Finally, the proportion of carboxyfluorescein succinimidyl ester (CFSE)^+^ cells in the dLNs was detected by flow cytometry. The in vivo CXCR2 activity was inhibited by intraperitoneal injection of 200 μl of SB225002 (50 μg/200 μl, a selective nonpeptide antagonist of CXCR2; MCE) into each mouse 1 h prior to DC injection [46]. The in vivo NF-κB activity was inhibited by intraperitoneal injection of 200 μl of QNZ (20 μg/200 μl; TopScience) into each mouse 1 h prior to each DC injection. All tests were repeated three times. All of the gating strategy and representative diagram are placed in Supplementary Fig. 2 g- h.

### Real-time quantitative reverse transcriptase polymerase chain reaction

RNA was extracted from DCs (3 × 10^5^/ml) treated with TCL (3 × 10^5^ cells), DP7-C (10 μg/ml) + TCL (3 × 10^5^/cells), TCL (3 × 10^5^ cells) + LPS (1 μg/ml) + CpG (10 μg/ml) + IFN-γ (50 ng/ml), or DP7-C (10 μg/ml) + TCL (3 × 10^5^ cells) + LPS (1 μg/ml) + CpG (10 μg/ml) + IFN-γ (50 ng/ml), DP7-C (10 μg/ml), calcitriol (10 nM), Tauro (50 μM), 16(R)-HETE (0.5 μM), acetylcholine (1 μM), PA (1 mM), and α-D-glucose (15 μM) for 24 h with RNA isolator total RNA extraction reagent (Vazyme Biotech Co., Ltd.). After reverse transcription of the extracted RNA with HiScript II Q RT SuperMix for qPCR (Vazyme Biotech Co., Ltd), the cDNA was used for mouse IL-1β, IL-10, IL-12p40, chemokine and chemokine receptor expression analysis according to the manufacturer’s instructions. Expression analysis according to the manufacturer’s instructions. The related primers are shown in Supplementary Table 1.

RNA was extracted from DCs treated with PBS, DP7-C (10 μg/ml), and PA (1 mM) for 24 h with RNA isolator total RNA extraction reagent (Vazyme Biotech Co., Ltd.). After reverse transcription of the extracted RNA with the miRNA 1st Strand cDNA Synthesis Kit (Vazyme Biotech Co., Ltd), the cDNA was used for miRNA expression analysis according to the manufacturer’s instructions. The expression levels of the miRNAs were normalized to the U6 level in each sample. The sequences of the primers used are shown in Supplementary Table 2.

### Transcriptomic sequencing

DCs (1 × 10^7^) were treated with PBS or DP7-C (10 μg/ml) for 4 h (*n* = 3), and then the residual drug was washed away, and the cells were lysed with TRIzol (Invitrogen). Finally, the samples were sent to Novogene (Beijing, China) for transcriptomic sequencing using an Illumina Novaseq 6000 system. Once the company completed quality control, sequencing and data analysis, Novomagic was used for subsequent data processing.

### Metabolomics sequencing

DCs (1 × 10^7^) were treated with PBS or DP7-C (10 μg/ml) for 24 h (*n* = 3). Then, the suspended cells were collected and washed three times with precooled PBS. The cell pellet was placed in liquid nitrogen for 15 min and stored at − 80 °C. Next, the adherent cells were collected, washed three times with precooled PBS, fixed with precooled 60% chromatographic-grade methanol for 5 min, and gently scraped off with a cell scraper. After centrifugation, they were quickly frozen in liquid nitrogen for 15 min and stored at − 80 °C. Finally, the samples were sent to Novogene (Beijing, China) for metabolomics sequencing using LC-MS/MS. Once the company completed quality control, sequencing and data analysis, Novomagic was used for subsequent data processing.

### miRNA sequencing

DCs (1 × 10^7^) were treated with PBS or DP7-C (10 μg/ml) for 24 h (*n* = 3), and then the residual drug was washed away, and the cells were lysed with TRIzol. Finally, the samples were sent to Novogene (Beijing, China) for miRNA sequencing using IIIumina SE50 system. Once the company completed quality control, sequencing and data analysis, Novomagic was used for subsequent data processing.

### Western blot analysis

Total protein was extracted from DCs treated with PBS, DP7-C (10 μg/ml) or PA (1 mM) for 24 h. Total protein was extracted from DCs transfected with Lipo3000 (9.6 μg)/miRNA negative control (miNC, 4.8 μg) or Lipo3000 (9.6 μg)/miR-142a-3p mimics (miR-142a-3p: UGUAGUGUUUCC UACUUUAUGGA, 4.8 μg; GenePharma, Shanghai, China) for 24 h, and total protein was extracted from DCs treated with DP7-C (10 μg/ml), PA (1 mM) or SB225002 (1 μM for 1 h) + DP7-C (10 μg/ml), or SB225002 (1 μM for 1 h) + PA (1 mM) for 24 h. The protein lysates were subjected to sodium dodecyl sulfate polyacrylamide gel electrophoresis and transferred to membranes. Then, the membranes were probed with antibodies against GAPDH, CXCR2, TAB2, NF-κB p65, NF-κB p-p65, and MMP14 (CST, USA) and incubated with horseradish peroxidase (HRP)-conjugated secondary antibody (Abcam, USA). Finally, a chemiluminescence system (Millipore, Massachusetts, USA) was used to visualize and photograph the target protein bands.

### In vivo immunization and cancer immunotherapy studies

To verify whether DP7-C or PA can enhance the antitumor effect of a DC vaccine loaded with HOCl-oxidized TCL, the mice were divided into three groups (PBS, DCs (3 × 10^5^/ml) loaded with TCL (3 × 10^5^ cells/ml) + LPS (1 μg/ml) + CpG (10 μg/ml) + IFN-γ (50 ng/ml), and DP7-C (10 μg/ml) or PA (1 mM) + DCs (3 × 10^5^/ml) loaded with TCL (3 × 10^5^ cells/ml) + LPS (1 μg/ml) + CpG (10 μg/ml) + IFN-γ (50 ng/ml)), each containing 5 or 6 mice. On day 0, LL2 tumor cells (3 × 10^5^/100 μl/mouse) were intravenously injected into the mice. On days 4, 11 and 18, DCs (2 × 10^6^/mouse) incubated with the above-indicated treatments for 24 h were intradermally injected into the mice in each group. The mice were sacrificed on day 21, the lungs were weighed and photographed, and the number of nodules on the lung surface was counted. The body weights were recorded every 2 days.

### Histological analysis

Main organs were harvested, fixed immediately using 4% paraformaldehyde and embedded in paraffin after the mice were sacrificed. The embedded tissue sections were dewaxed, rehydrated and then staining with Mayer’s hematoxylin and eosin (H&E) stain according to the vendor’s instructions (Solarbio, China).

### Statistical analysis

All data were quantified and plotted using GraphPad Prism 8.0 with one-way ANOVA with multiple comparisons tests and are presented as the means ± SEM. *p value < 0.05, **p value < 0.01 and ***p value < 0.001 were considered to be statistically significant.

## Supplementary Information

**Additional file 1: Table S1.** The detected cytokines, chemokines, chemokine receptors and their primers. **Table S2.** The detected microRNAs and their primers. **Figure S1.** DP7-C/TCL-DCs do not enhance the antitumor effect of DC vaccines. **Figure S2.** The antigen uptake and presentation efficiency of DCs. **Figure S3.** The maturation of DCs and the secretion of cytokines by DCs were detected. **Figure S4.** Metabolomics sequencing results of DP7-C-treated DCs. **Figure S5.** Gene expression analysis of metabolite-treated DCs. **Figure S6.** HE staining-based analysis of major organs from each treatment group. **Figure S7.** The effect of vaccine formulation on DC toxicity and DC polarization.

## Data Availability

The datasets generated and/or analyzed during the current study are available from the corresponding author upon reasonable request.

## Abbreviations

α-D-Glucose: α-D-glucose-1,6-bisphosphate
BMDCs: Bone marrow-derived DCs
CFDA-SE: Carboxyfluorescein diacetate succinimidyl ester
DC: Dendritic cell
dLNs: draining lymph nodes
DMEM: Dulbecco’s modified Eagle’s medium
DP7-C: Cholesterol modified VQWRIRVAVIRK
DPBS: Dulbecco’s phosphate buffered saline
FBS: Fetal bovine serum
FITC: Fluorescein isothiocyanate
GM-CSF: Granulocyte-macrophage colony-stimulating factor
HOCl: Hypochlorous acid
HOCl-TCL: HOCl-oxidized TCLs
LC: Langerhans cell
LCI: LPS + CpG + IFN-γ
LNs: Lymph nodes
LPS: Lipopolysaccharide
Mo-DC: Monocyte derived DC
miRNA: microRNA
PA: Pantothenic acid
PS: Streptomycin and penicillin
RPMI: Roswell Park Memorial Institute
Tauro: Taurochenodeoxycholic acid
TCLs: Tumor cell lysates
