# Supplementary material for: Cholesterol modified DP7 and pantothenic acid induce dendritic cell homing to enhance the efficacy of dendritic cell vaccines
Source: Mol Biomed. 2021 Dec 5;2:37. doi: 10.1186/s43556-021-00058-9 (PMC8643384; doi:10.1186/s43556-021-00058-9)
Supplement: Supplementary file 1 — Additional file 1: Table S1. The detected cytokines, chemokines, chemokine receptors and their primers. Table S2. The detected microRNAs and their primers. Figure S1. DP7-C/TCL-DCs do not enhance the antitumor effect of DC vaccines. Figure S2. The antigen uptake and presentation efficiency of DCs. Figure S3. The maturation of DCs and the secretion of cytokines by DCs were detected. Figure S4. Metabolomics sequencing results of DP7-C-treated DCs. Figure S5. Gene expression analysis of metabolite-treated DCs. Figure S6. HE staining-based analysis of major organs from each treatment group. Figure S7. The effect of vaccine formulation on DC toxicity and DC polarization. [file 43556_2021_58_MOESM1_ESM.doc]

**DP7-C and Pantothenic acid induces homing of dendritic cells to enhance the efficacy of dendritic cell vaccine**

Running title：DP7-C and PA induces homing of dendritic cells

Rui Zhang^1†^, Lin Tang^1†^, Qing Li^1^, Yaomei Tian^1^, Binyan Zhao^1^, Bailing Zhou^1^, Li Yang^1^*

^1^State Key Laboratory of Biotherapy and Cancer Center, West China Hospital, Sichuan University, and Collaborative Innovation Center for Biotherapy, Chengdu, 610041, China.

Corresponding Author: Li [Yang; State Key Laboratory of Biotherapy and Cancer Center, West China Hospital, Sichuan University, Chengdu 610041, People’s Republic of China; Email: yl.tracy73@gmail.com](mailto:Yang Li; State Key Laboratory of Biotherapy and Cancer Center, West China Hospital, Sichuan University, Chengdu 610041, People's Republic of China;                        Email: yl.tracy73@gmail.com); Tel: 18628182400

**Materials and methods**

**Preparation of FITC labeled tumor cell lysates (TCL-FITC)**

Two mg of FITC in 1 mL of 20 mmol/L carbonate buffer (pH 9.5) was added to a solution of TCL (1 mg/mL, 10 mL). The solution was incubated with continuous stirring at 4 °C for 18 h in the dark. The reaction mixture was dialyzed against distilled water (MWCO 1000) to obtain TCL-FITC.

**CCK-8 assay**

DCs (5 × 10^4^ cells) were separately seeded in 96-well plates. Then, the cells were incubated with TCL (3×10^5^ cells) + LPS (1 μg/ml) + CpG (10 μg/ml) + IFN-γ (50 ng/ml), DP7-C (10 μg/ml) + TCL (3×10^5^ cells) + LPS (1 μg/ml) + CpG (10 μg/ml) + IFN-γ (50 ng/ml), or PA (1 mM) + TCL (3×10^5^ cells) + LPS (1 μg/ml) + CpG (10 μg/ml) + IFN-γ (50 ng/ml) in 100 μL of 1640 medium for 24 h. Subsequently, 10 μL of CCK-8 solution was added to each well and incubated for 4 h at 37 °C. Next, the absorbance of each sample at 450 nm was measured using a microplate reader (Bio-Rad), and the untreated cells were considered as controls.

**Detection of DP7-C and PA-induced DC polarization**

To detect DC polarization, DCs (3×10^5^/ml) were incubated with TCL (3×10^5^ cells) + LPS (1 μg/ml) + CpG (10 μg/ml) + IFN-γ (50 ng/ml), or DP7-C (10 μg/ml) + TCL (3×10^5^ cells) + LPS (1 μg/ml) + CpG (10 μg/ml) + IFN-γ (50 ng/ml) or PA (1 mM) + TCL (3×10^5^ cells) + LPS (1 μg/ml) + CpG (10 μg/ml) + IFN-γ (50 ng/ml) for 24 h and then stained with anti-mouse CD11c, anti-mouse MHCII, anti-mouse-CD8α, anti-mouse-XCR1, anti-mouse CD11b, anti-mouse CD172A, anti-mouse CX3CR1, and anti-mouse CD205 antibody (BD, US) for 40 min, followed by flow cytometry. All tests were repeated three times.

Table S1. The detected cytokines, chemokines, chemokine receptors and their primers.

|  | Forward | Reverse |
| --- | --- | --- |
| IL-1β  IL-10  IL-12p40  CXCR2 | GCTTCAGGCAGGCAGTATCA  CAGAGAAGCATGGCCCAGAA  CCTGTGACACGCCTGAAGAA  GGGTCGTACTGCGTATCCTG | AATGGGAACGTCACACACCA  GCTCCACTGCCTTGCTCTTA  GTGAGTGGCTCAGAGTCTCG  AGACAAGGACGACAGCGAAG |
| CCL5 | CCTCACCATATGGCTCGGAC | ACGACTGCAAGATTGGAGCA |
| CXCL3 | CCATCCAGAGCTTGACGGTG | TGGGGGTTGAGGCAAACTTC |
| CCR7 | GACACGCTGAGATGCTCACT | GACTACCACCACGGCAATGA |
| CCL22 | CAGGTCCCTATGGTGCCAAT | CTAAACGTGATGGCAGAGGGT |
| CCR4 | CGCCATCCAGGCTACAGAAA | GCGTGTAAGAGGAGCTGGAC |
| CX3CL1 | GGCTACTGGCTTTCCTTGGT | TAGCGGAGGCCTTCTACCAT |
| CCR2 | CACCCTGTTTCGCTGTAGGA | CATGGCCTGGTCTAAGTGCT |
| CXCL12 | CGGTTCTTCGAGAGCCACA | TTGTTCTTCAGCCGTGCAAC |
| CCL17 | GACCTTCACCTCAGCTTTTGG | GGCATGCTGCAGAAAAGTCC |
| CCL12 | ACCACCATCAGTCCTCAGGTA | GGACACTGGCTGCTTGTGAT |
| CXCR5 | CTAGCCATCGTCCATGCTGT | GAGTTCCGGTAAGGCGAACA |
| CCL2 | AGGTGTCCCAAAGAAGCTGT | GACCTTAGGGCAGATGCAGTT |
| CCL3 | CCGGAAGATTCCACGCCAAT | TCAGGCATTCAGTTCCAGGT |
| CCL19 | CCTGCTGGTTCTCTGGACCT | CGGAAGGCTTTCACGATGTTC |
| CXCL1 | CGCCTATCGCCAATGAGCTG | TGAGGGCAACACCTTCAAGC |
| XCL1 | GCCAAATGGGTGAAAGCAGC | TACCCAGTCAGGGTTATCGC |
| CCRL2 | GCCCCGGACGATGAATATGA | CACGTTGTCCAAGAGACCCA |
| CXCR4 | CCGGTACCTCGCTATTGTCC | TCCACAGGCTATCGGGGTAA |
| CX3CR1 | ACCTCCTTCCCTGAACTGGA | CAGACCGAACGTGAAGACGA |
| CCL21 | GTTTAGGCTGTCCCATCCCG | AGACTTAGAGGTTCCCCGGT |
| Plexin-A4 | GCACCAACCCTCGTATCACA | ATTCCACACCAGCCACTTTCA |
| PGE2 | CACGCGTGTACCTATTTCGC | GGCGCCTGTAGAAGTAAGGG |
| SIP | GAGCCAGAAGTGGCAGTCAT | GGAATGGCTCTCAGCAATCG |
| CLEC-2 | AAACATCAAGCCCCGGAAAC | ATTGTTGCAGAGTCGCTGAGA |
| Sema3A | GTCTTCCGGGAACCAACAAC | TGCACAGGCTTTGCCATAGA |
| SIPR1 | GGAGGTTAAAGCTCTCCGCA | AATGCCATGGTCCTTCTCCG |
| IL-18 | CGACTTCACTGTACAACCGC | GGGGTTCACTGGCACTTTGA |
| CDC42 | GTATGTGGAGTGCTCCGCCC | CTCTTCTTCGGTTCTGGAGGC |
| CySL | GGAACTGAAAATCTGACGACATC | CCACACGGAGAGGCAATGTA |
| DOCK8 | AGATGGAGCAGTGTGCTGAC | TGCTTTGCCTACCAGGGATG |
| β-actin | TGTGCTGTCCCTGTATGCCTCT | GGAACCGCTCGTTGCCAATAGT |

Table S2. The detected microRNAs and their primers.

|  | U6 | miR142a-3p |
| --- | --- | --- |
| Forward | CTCGCTTCGGCAGCACA | GCGCGTGTAGTGTTTCCTACTT |
| Revise | AACGCTTCACGAATTTGCG | AGTGCAGGGTCCGAGGTATT |
| RT | AACGCTTCACGAATTTGCGT | GTCGTATCCAGTGCAGGGTCCGAGGTATTCGCACTGGATACGACTCCATA |


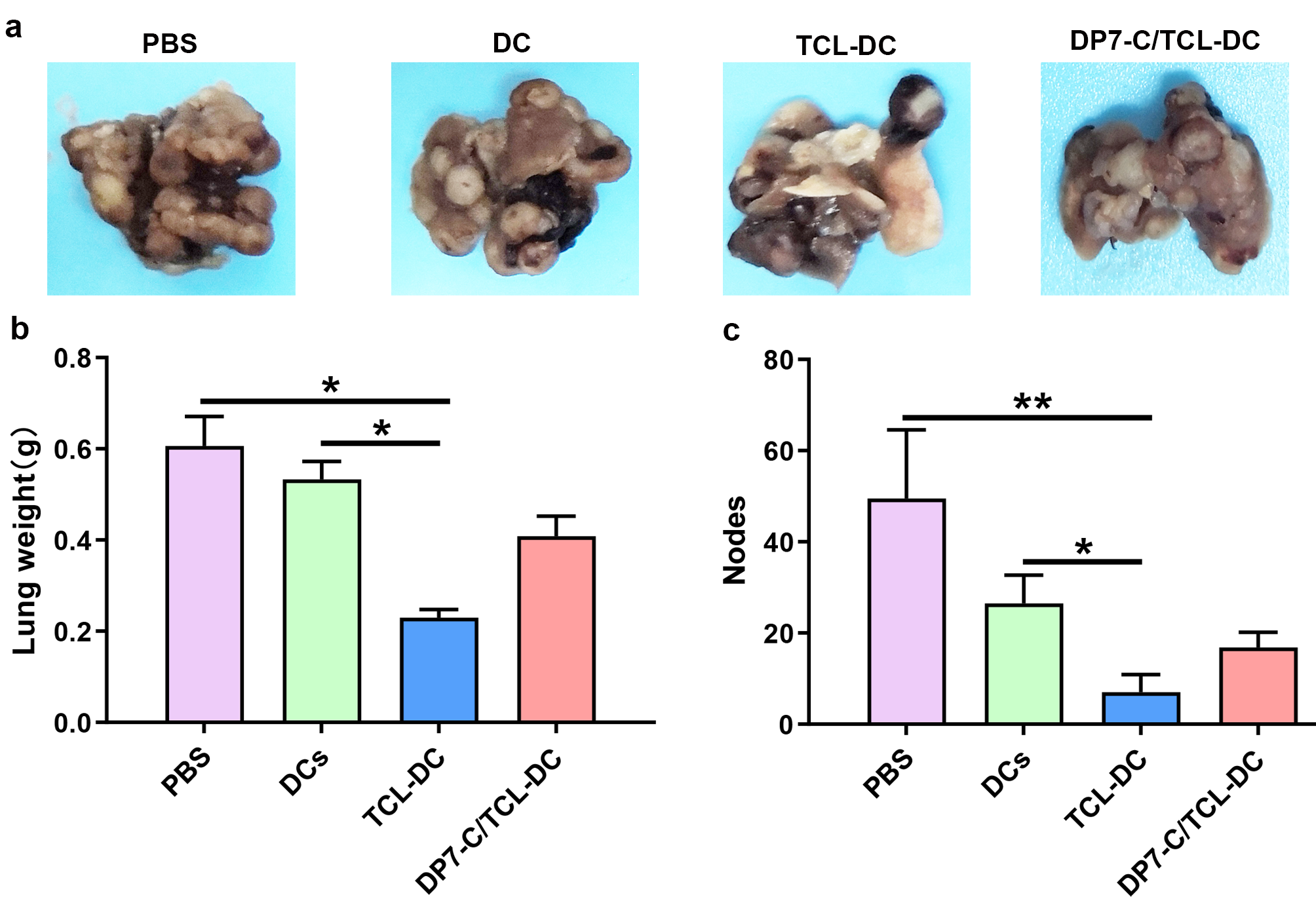


Figure S1. DP7-C/TCL-DCs do not enhance the antitumor effect of DC vaccines. a. Representative results of metastatic lung nodules. b. Lung weight. c. Number of pulmonary nodules. Significance was calculated using a one-way ANOVA with multiple comparisons tests (**p* < 0.05, ***p* < 0.01).


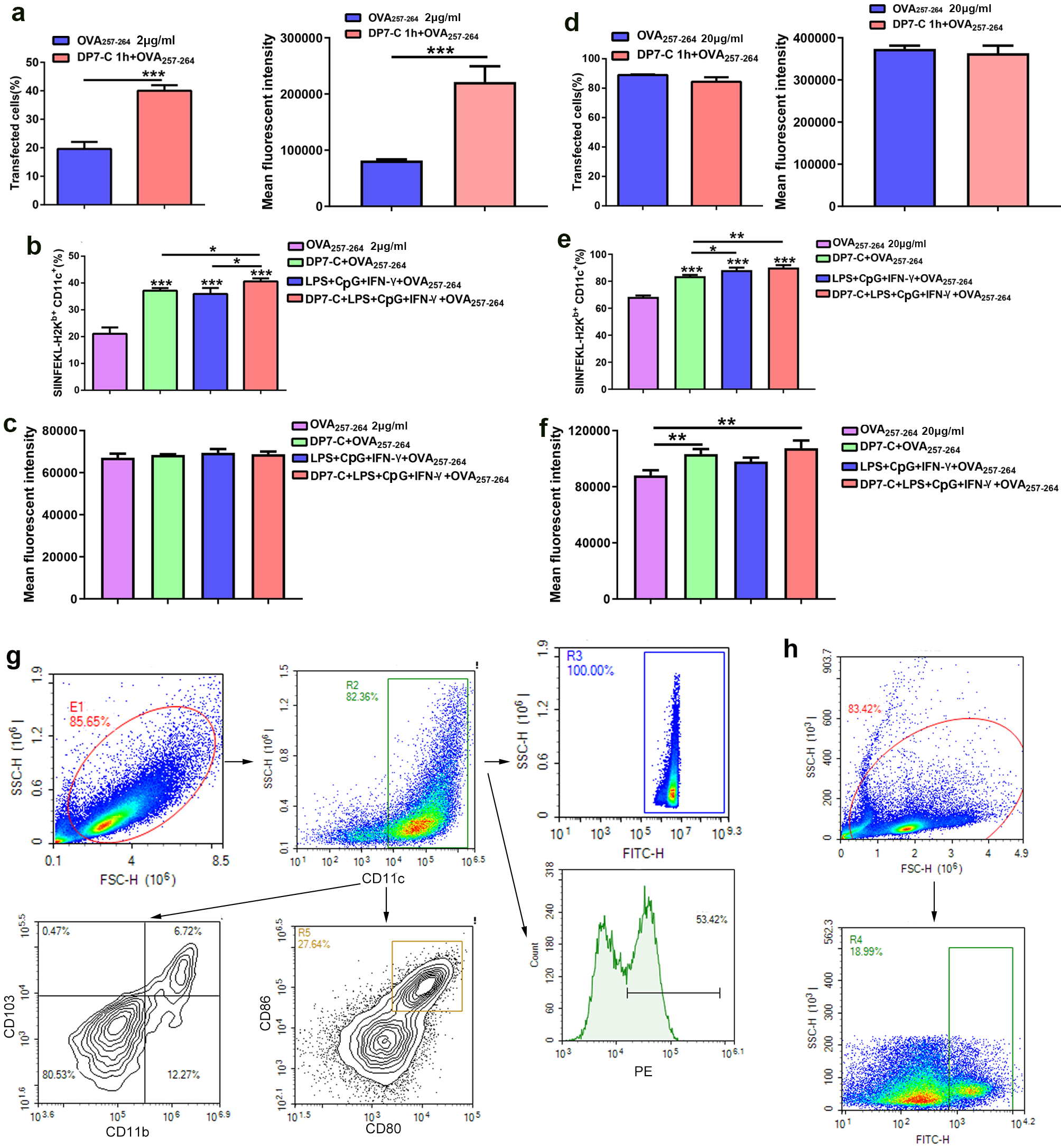


Figure S2. The antigen uptake and presentation efficiency of DCs. a, d. The OVA_257-264_ uptake efficiency of DCs and mean fluorescence intensity statistics of DC antigen uptake. b, e. The antigen presentation efficiency of BMDCs stained with the monoclonal antibody 25-D1.16, which recognizes the OVA_257-264_-H-2Kb complex. c, f. Mean fluorescence intensity statistics of DC antigen presentation. g. The gating strategy of DC uptake efficiency, antigen presentation efficiency, DC maturation ratio and CD103^+^ DC ratio and their representative dot plot (FACS). h. The gating strategy for the detection of the efficiency of DC migration to LNs and representative dot plot (FACS). Significance was calculated using a one-way ANOVA with multiple comparisons tests (**p* < 0.05, ***p* < 0.01, ****p* < 0.001).


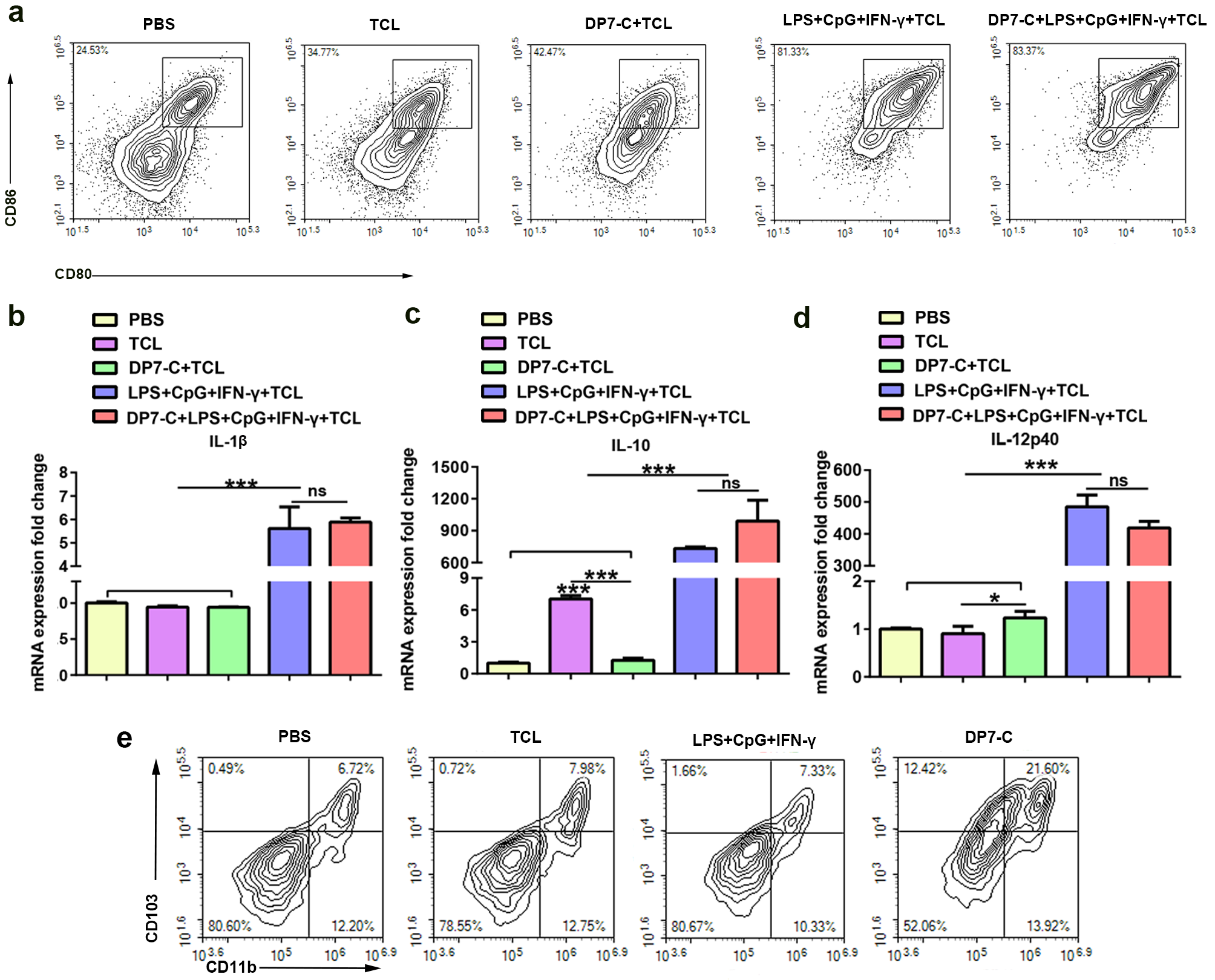


Figure S3. The maturation of DCs and the secretion of cytokines by DCs were detected. a. The percentages of mature DCs among all DCs after different treatments. b-d. The expression of IL-1β, IL-10 and IL-12p40 was detected. e. The percentages of CD103^+^ DCs among all DCs after different treatments. Significance was calculated using a one-way ANOVA with multiple comparisons tests (**p* < 0.05, ****p* < 0.001).


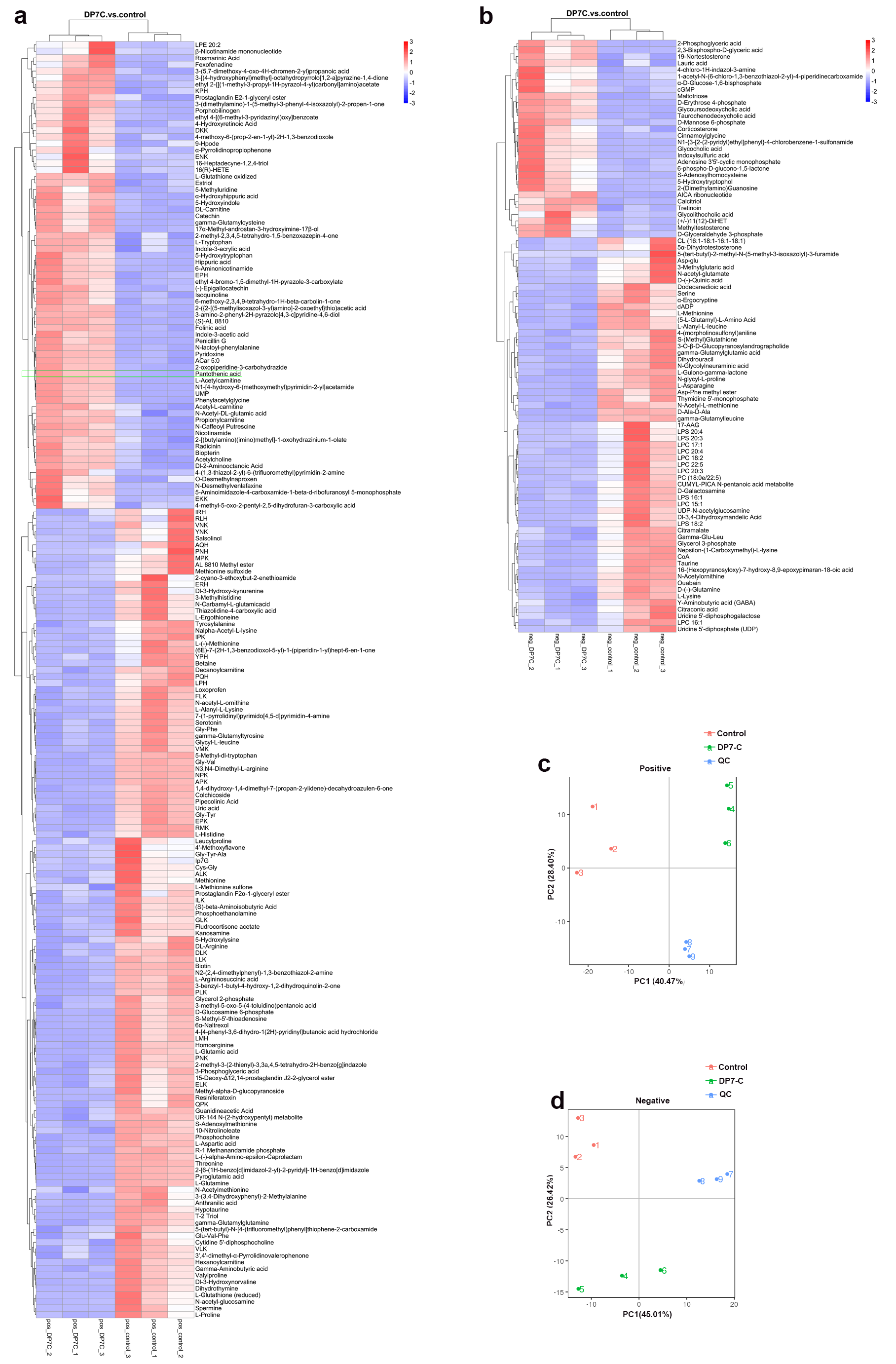


Figure S4. Metabolomics sequencing results of DP7-C-treated DCs. a. Cluster analysis of different metabolites in positive ion mode. b. Cluster analysis of different metabolites in negative ion mode. c. PCA of different metabolites in positive ion mode. d. PCA of different metabolites in negative ion mode.


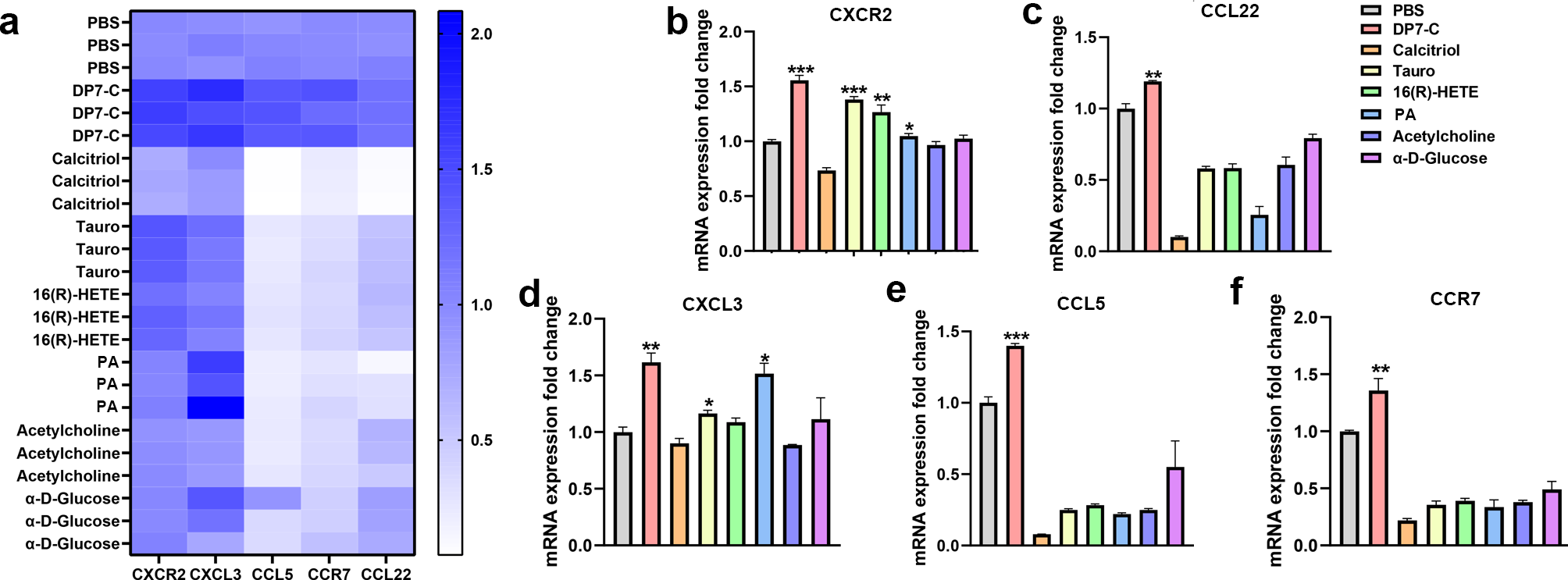


Figure S5. Gene expression analysis of metabolite-treated DCs. a. Heat map of gene expression. b. CXCR2 expression analysis. c. CCL22 expression analysis. d. CXCL3 expression analysis. e. CCL5 expression analysis. f. CCR7 expression analysis. Significance was calculated using a one-way ANOVA with multiple comparisons tests (**p* < 0.05, ***p* < 0.01, ****p* < 0.001).


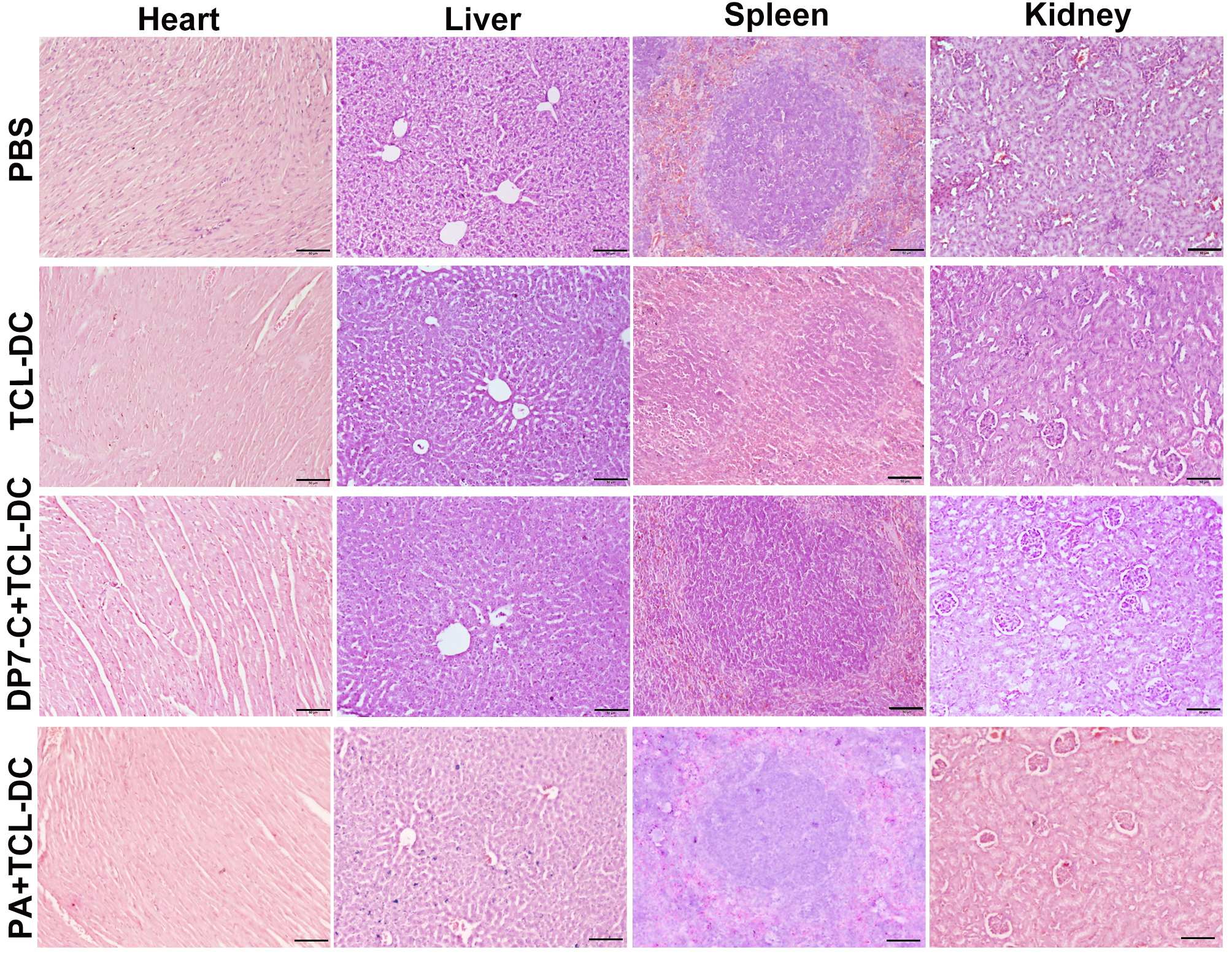


Figure S6. HE staining-based analysis of major organs from each treatment group. Scale bar, 50 μm.


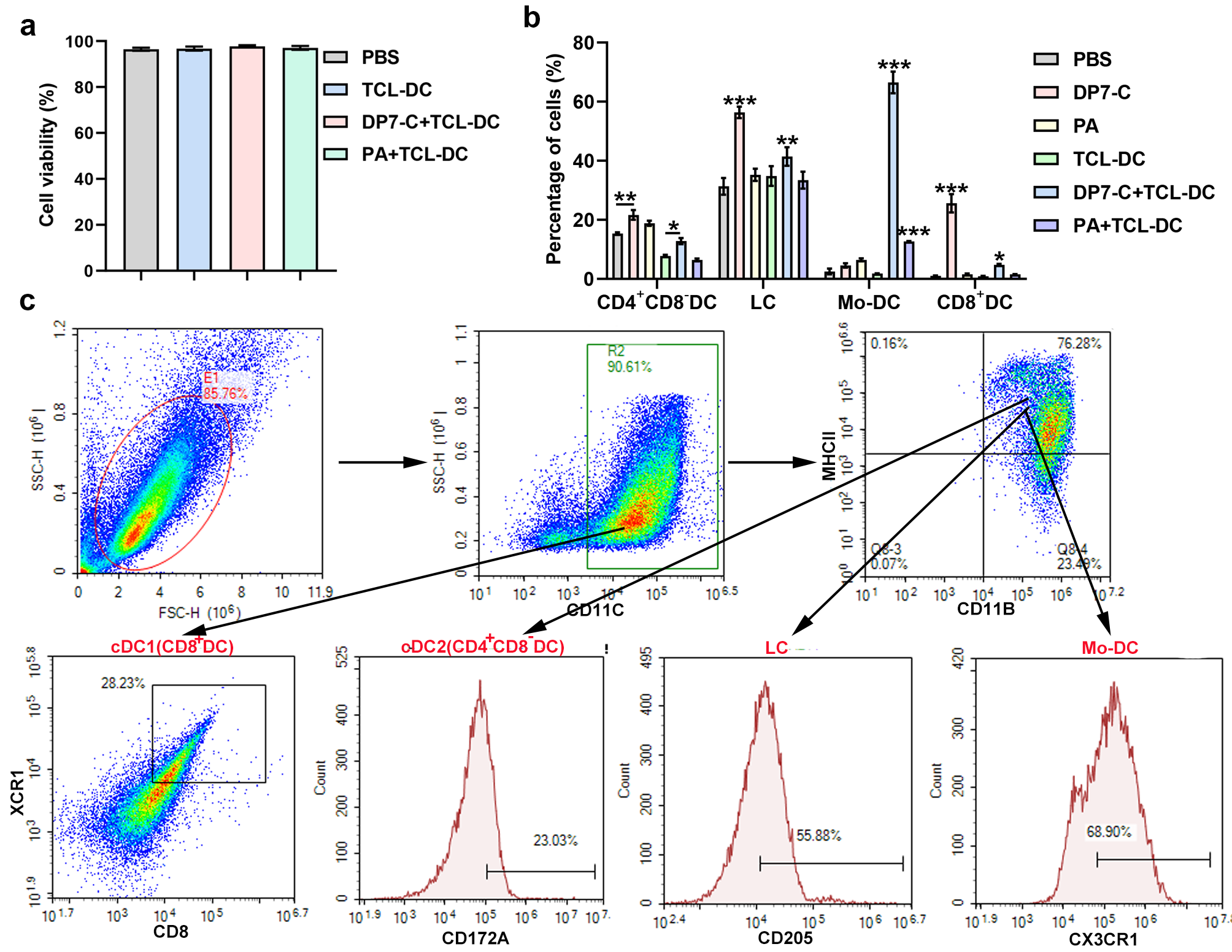


Figure S7. The effect of vaccine formulation on DC toxicity and DC polarization. a. CCK-8 assay of TCL-DC, DP7-C+TCL-DC and PA+TCL-DC. b. Polarization analysis of DC treated with PBS, DP7-C, PA, TCL, DP7-C+TCL and PA+TCL. Langerhans cell (LC), Monocyte derived DC (Mo-DC).
